# Flavokawain B and Doxorubicin Work Synergistically to Impede the Propagation of Gastric Cancer Cells via ROS-Mediated Apoptosis and Autophagy Pathways

**DOI:** 10.3390/cancers12092475

**Published:** 2020-09-01

**Authors:** You-Cheng Hseu, Ruei-Wan Lin, Yi-Chun Shen, Kai-Yuan Lin, Jiunn-Wang Liao, Varadharajan Thiyagarajan, Hsin-Ling Yang

**Affiliations:** 1 Department of Cosmeceutics, College of Pharmacy, China Medical University, Taichung 40402, Taiwan; ychseu@mail.cmu.edu.tw; 2 Department of Health and Nutrition Biotechnology, Asia University, Taichung 41354, Taiwan; 3 Chinese Medicine Research Center, China Medical University, Taichung 40402, Taiwan; 4 Research Center of Chinese Herbal Medicine, China Medical University, Taichung 40402, Taiwan; 5 Institute of Nutrition, China Medical University, Taichung 40402, Taiwan; ann30245@gmail.com (R.-W.L.); u104076012@cmu.edu.tw (Y.-C.S.); 6 Department of Medical Research, Chi Mei Medical Center, Tainan 71004, Taiwan; d84306@yahoo.com.tw; 7 Department of Biotechnology, Chia Nan University of Pharmacy and Science, Tainan 71004, Taiwan; 8 Graduate Institute of Veterinary Pathology, National Chung-Hsing University, Taichung 402, Taiwan; jwliao@dragon.nchu.edu.tw

**Keywords:** flavokawain B, doxorubicin, apoptosis, autophagy, ROS

## Abstract

**Simple Summary:**

Among various kinds of treatment strategies for cancers, combination therapy has attracted significant attention due to its beneficial effects than the individual effects of the same compounds. Based on this idea, this study has investigated the synergistic effects of combination treatment of a natural anti-cancer agent flavokawain B (FKB) and a chemotherapeutic agent Doxorubicin on human gastric cancer cells and the underlying molecular mechanisms were deciphered through in vitro and in vivo approaches. Experimental data obtained in this study provided promising application prospects of FKB + Doxrubicin combination treatment in human gastric cancer cells.

**Abstract:**

Chalcone flavokawain B (FKB) possesses a chemopreventive and anti-cancer activity. Doxorubicin is a chemotherapeutic DNA intercalating agent widely used in malignancy treatment. The present study investigated whether synergistic effects exist between the combination of FKB (1.25–5 µg/mL) and doxorubicin (0.5 µg/mL) on the apoptosis and autophagy in human gastric cancer (AGS) cells, and the possible in vitro and in vivo mechanisms. The MTT assay measured cell viability. Various apoptotic-, autophagy-associated protein expression was determined by the Western blot technique. FKB+doxorubicin synergy was estimated by the Chou-Talalay combination index (CI) method. In vivo studies were performed on BALB/*c* mice. Results showed that compared to FKB/doxorubicin treatments, low doses of FKB+doxorubicin suppressed AGS cell growth. FKB potentiated doxorubicin-induced DNA fragmentation, apoptotic cell death, and enhanced doxorubicin-mediated mitochondrial, death receptor pathways. FKB+doxorubicin activated increased LC3-II accumulation, p62/SQSTM1 expression, and AVO formation as compared to the FKB/doxorubicin alone treatments indicating autophagy in these cells. The death mechanism in FKB+doxorubicin-treated AGS cells is due to the activation of autophagy. FKB+doxorubicin-mediated dysregulated Bax/Bcl-2, Beclin-1/Bcl-2 ratios suggested apoptosis, autophagy induction in AGS cells. FKB+doxorubicin-induced LC3-II/AVOs downregulation was suppressed due to an apoptotic inhibitor Z-VAD-FMK. Whereas, 3-methyladenine/chloroquine weakened FKB+doxorubicin-induced apoptosis (decreased DNA fragmentation/caspase-3). Activation of ERK/JNK may be involved in FKB+doxorubicin-induced apoptosis and autophagy. FKB+doxorubicin-triggered ROS generation, but NAC attenuated FKB+doxorubicin-induced autophagic (LC3 accumulation) and apoptotic (caspase-3 activation and PARP cleavage) cell death. FKB+doxorubicin blocked gastric cancer cell xenografts in nude mice in vivo as compared to FKB/doxorubicin alone treatments. FKB and doxorubicin wielded synergistic anti-tumor effects in gastric cancer cells and is a promising therapeutic approach.

## 1. Introduction

Gastric cancer is a common malignant cancer and the second leading cause of death around the world. Data from the cancer registry annual report of Taiwan has indicated that gastric cancer is a leading cause of cancer deaths in Taiwan [1]. Due to its asymptomatic nature, this cancer type is being diagnosed only at the advanced stages frequently leading to deaths. Chemotherapy is the most commonly used method to treat this cancer type. However, this treatment method also showed certain side effects, such as hepatotoxicity, immunosuppression, myelosuppression, etc. [2]. Therefore, effective chemotherapeutic agents from the herbal origin would be a treatment strategy in the management of gastric cancer. 

Apoptosis is a complex process involved in maintaining homeostasis in multicellular organisms. Programmed cell death or apoptosis uses a complex series of biochemical events that causes the characteristic cell morphology and death. Apoptosis has the following features: Stereotyped morphology showing chromatin condensation, internucleosomal DNA cleavage, and the activation of a family of cysteine-aspartic acid proteases (caspases) [3,4]. Therefore, chemical or biological agents that were reported to cell-cycle arrest and induction of apoptosis could be a promising intervention in the management of cancer [5].

Cellular self-digestion or autophagy is characterized as a dynamic process that is involved in changing long-lived proteins and organelles. This process recycles biological materials. This process is also essential for cell components to maintain their quality [6,7] and can lead to type II programmed cell death when used excessively, because they can intensely degrade the mitochondria and other survival molecules [8]. Autophagic roles in the treatment of cancer in the literature have clarified this issue and, further, these studies show that it is possible to suppress autophagic cell death in malignant tumors. Anti-cancer drugs and treatments often stimulate autophagy and not apoptosis. Apoptosis should be considered an alternative pathway for cancer cell death [9,10]. Microtubule-related light chain 3 (LC3), ATG5, ATG7, mammalian target of rapamycin (mTOR), ROS, and beclin-1 are atomic and cell signaling pathways that have been suggested to control autophagy [6,11]. Both processes have the same complex functional relationship and are dependent on cellular context. Research shows that too much ROS can severely damage DNA and proteins; it also weakens the mitochondrial membrane potential (∆Ψm), which leads to autophagy and apoptosis [12,13]. 

Doxorubicin (Dox) is a first line therapy for numerous cancers whether alone or in combination with another chemotherapeutic treatment. Doxorubicin has the following mechanisms: Intercalation into DNA disrupting gene expression, generation of reactive oxygen species, and the inhibition of topoisomerase II [14]. Doxorubicin is shown to increase intracellular ceramide levels that result in the release of CREB3L1 (a membrane transcription factor) [15]. It involves the expression of various genes, but more importantly the tumor suppressor, p21 is expressed. Tumor cells characterized by elevated CREB3L levels show a sensitivity to doxorubicin, but decreased levels show resistance [16]. Despite being used as a antineoplastic agent, doxorubicin is associated with cardiotoxicity and resistance [17,18]. Previous studies supported the fact that the use of a combination treatment strategy by combining doxorubicin with another agent would synergistically inhibit cancer cell proliferation and induction of ROS-mediated autophagic cell death in different cancer cell types, thus reducing the dosage requirement of Dox [19,20]. 

Chalcones belong to the flavonoids family and are natural compounds extracted from the roots of kava-kava plant. They also exhibit various anti-cancer, anti-inflammatory, and anti-nociceptive properties [21]. Previous research has suggested that kava chalcones (at the dosage of 50 mg/kg) could effectively suppress the in vivo growth rate of RT4 cells in the athymic nude mice without any toxicity [22]. Flavokawain B (FKB) from among all chalcones has shown a characteristic that demonstrates the growth inhibitory effect for various cancers via several signaling pathways [23]. It has been shown that FKB isolated from the rhizomes of *Alpinia pricei* Hayata was able to induce apoptosis in oral carcinoma (HSC-3) [24]. However, in a recent study, we have also shown that FKB from *Alpinia pricei* Hayata caused ROS-mediated apoptotic and autophagic cell death in human lung adenocarcinoma (A549) cells [25]. 1,3-diaryl-2-propen-1-ones are important organic mixes that are found in edible. These chalcones have anti-cancer, anti-fungal, anti-microbial, anti-tumor, calming, and cytotoxic activities [26]. 

The chalcone FKB induces (ROS-mediated) autophagy cell death (not apoptosis) in AGS cells [27]. It has also been reported that doxorubicin induces apoptosis in various cancer cells, which is known to be mediated through ROS [28,29]. Therefore, this study was aimed to investigate the synergistic effects of FKB and doxorubicin combination treatment on AGS cells and the role of apoptosis and autophagy mechanisms were elucidated. 

## 2. Results

### 2.1. Combination Effects of FKB and Doxorubicin on AGS Cells 

The chalcone FKB induces ROS-mediated autophagic cell death (not apoptosis) in AGS cells [27]. Doxorubicin induces ROS-mediated apoptosis in various cancer cells [28,29]. The AGS cells were exposed to various concentrations of FKB 1.5–5 µg/mL and 0.5 µg/mL of doxorubicin for 24 h, respectively, to look into any potential effects as to the propagation and survival of ASG cells. Table 1 shows that FKB and doxorubicin treatments caused significant cell death in AGS cells. Notably, AGS cells treated with FKB (5 µg/mL) and doxorubicin (0.5 µg/mL) showed 64.4 ± 1.2 and 77.1 ± 3.7% cell viability. To determine the combination effect between FKB and doxorubicin, the concentration of FKB and doxorubicin in different ratios were tested. The predicted value and combination index (CI) for the combined effects of FKB and doxorubicin in the AGS cells were determined according to Chou and Talalay [30]. Table 1 shows the values of CI (0.64, 0.28, and 0.07 for a combination of 0.5 µg doxorubicin with 1.25, 2.5, and 5 µg/mL FKB, respectively) clearly demonstrate that the combination treatment wielded synergistic growth inhibition of AGS cells. As observed from the higher synergistic effects (low CI value), the combination of FKB and doxorubicin therapy was more effective on AGS cells.

In addition to AGS cells, we determined the effects of FKB and doxorubicin on other human gastric cancer cells (SCM-1 and MKN45). Table 2 and Table 3 indicate that FKB and doxorubicin treatments induced significant cell death in SCM-1 and MKN45. The values for CI (0.53, 0.62, and 0.67; 0.57, 0.70, and 0.59) for the combination of 0.5 µg doxorubicin with 1.25, 2.5, and 5 µg/mL FKB, respectively, showed that the combination treatment wielded synergistic growth inhibition of SCM-1 and MKN45.

Table 1. Flavokawain B (FKB) and doxorubicin co-treatment exhibited synergistic effects in human gastric cancer (AGS) cells. AGS cells were treated with increasing concentrations of FKB (0, 1.25, 2.5, or 5 µg/mL) and/or in combination with doxorubicin (0.5 µg/mL) for 24 h. Cell viability was determined using the MTT assay. After the incubation period, the effect of individual or co-treatments of both FKB and doxorubicin on AGS cell survival were determined according to the Chou and Talalay method. Low combination index (CI) values signify higher synergistic effects.

Table 2. FKB and doxorubicin co-treatment exhibited synergistic effects in human gastric cancer (SCM-1) cells. SCM-1 cells were treated with increasing concentrations of FKB (0, 1.25, 2.5, or 5 µg/mL) and/or in combination with doxorubicin (0.5 µg/mL) for 24 h. Cell viability was determined using the MTT assay. After the incubation period, the effect of individual or co-treatments of both FKB and doxorubicin on SCM-1 cell survival were determined according to the Chou and Talalay method. Low combination index (CI) values signify higher synergistic effects.

Table 3. FKB and doxorubicin co-treatment exhibited synergistic effects in human gastric cancer (MKN45) cells. MKN45 cells were treated with increasing concentrations of FKB (0, 1.25, 2.5, or 5 µg/mL) and/or in combination with doxorubicin (0.5 µg/mL) for 24 h. Cell viability was determined using the MTT assay. After the incubation period, the effect of individual or co-treatments of both FKB and doxorubicin on MKN45 cell survival were determined according to the Chou and Talalay method. Low combination index (CI) values signify higher synergistic effects.

### 2.2. Doxorubicin-Induced Apoptosis Is Potentiated by FKB Treatment in AGS Cells

**Figure 1:**
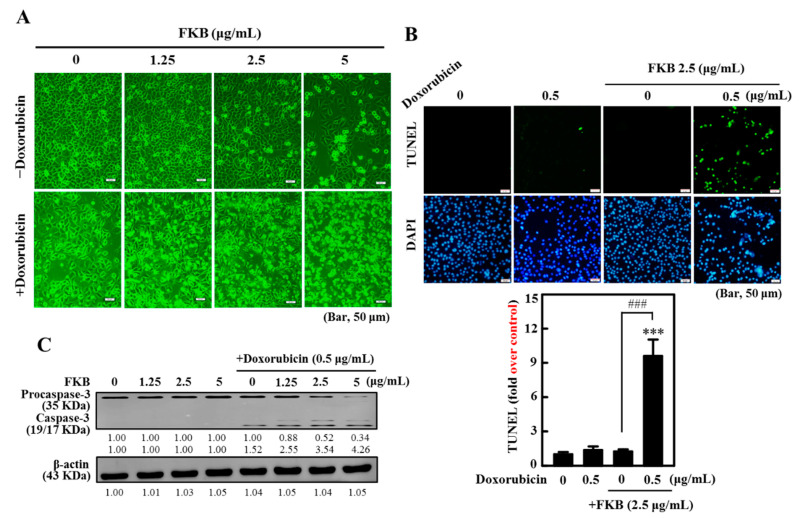
The synergistic effects of flavokawain B (FKB) and co-treatment with doxorubicin induced apoptosis of AGS cells in human gastric cancer. Cells have been exposed to FKB (0–2.5 μg/mL), doxorubicin (0.5 μg/mL), and a 24-h mixture of them. (**A**) Microscopy of phase-contrast (200 scale magnification) was used to investigate structural changes. (**B**) A TUNEL assay was conducted to determine the fragmentation of apoptotic DNA. The green florescence indicates the number of TUNEL-positive cells from three separate samples in the microscopic fields (Bar, 50 micron). (**C**) Western blot study of the protein content of caspase-3. It shows typical results of three independent experiments. On SDS-PAGE, the protein (50 μg) was resolved from each sample, and Western blot was performed. β-actin was used as an internal control of loads. Densitometric analysis calculated relative changes in protein bands with the control as 1.0-fold, as shown just below the gel results (Figure S1). Values were expressed as mean ± SD (*n* = 3). Significant at *** *p* < 0.001 compared to untreated control cells. Significant at ^###^ *p* < 0.001 compared to FKB-treated cells.

FKB (1.25–5 µg/mL) and doxorubicin (0.5 µg/mL) induced abrupt morphological changes (cell shrinkage) in AGS cells signifying the anti-tumor properties of FKB and doxorubicin through the inhibition of cell survival and induction of apoptosis (Figure 1A). A TUNEL assay was done and established FKB enhances apoptotic DNA fragmentation in AGS cells after treatment with doxorubicin. Fluorescence microscopy images revealed increased green fluorescence and TUNEL-positive cells from the doxorubicin treatment (0.5 µg/mL, 24 h). This further represents increased apoptotic DNA fragmentation (Figure 1B). Interestingly, the combination treatment (FKB+doxorubicin) showed a significant increase in DNA fragmentation (~12-fold) in AGS cells (Figure 1B), demonstrating that the FKB treatment significantly potentiates the doxorubicin-induced apoptosis in AGS cells. 

Caspase-3 (CPP32), a cytosolic protein, typically shows itself as a 32 KDa inactive precursor. This protein is cleaved proteolytically into a heterodimer at the time the cell starts apoptosis [31]. Figure 1C and Figure S1C show that the involvement of caspase-3 activation is also supported by Western blot analysis. This Western blot analysis shows that the synergistic effects of the combination of FKB and doxorubicin induced proteolytic cleavage of pro-caspase-3 into an active form, i.e., 19/17 KDa fragment.

### 2.3. FKB Enhanced Doxorubicin-Mediated Mitochondrial Cascades in AGS Cells 

**Figure 2:**
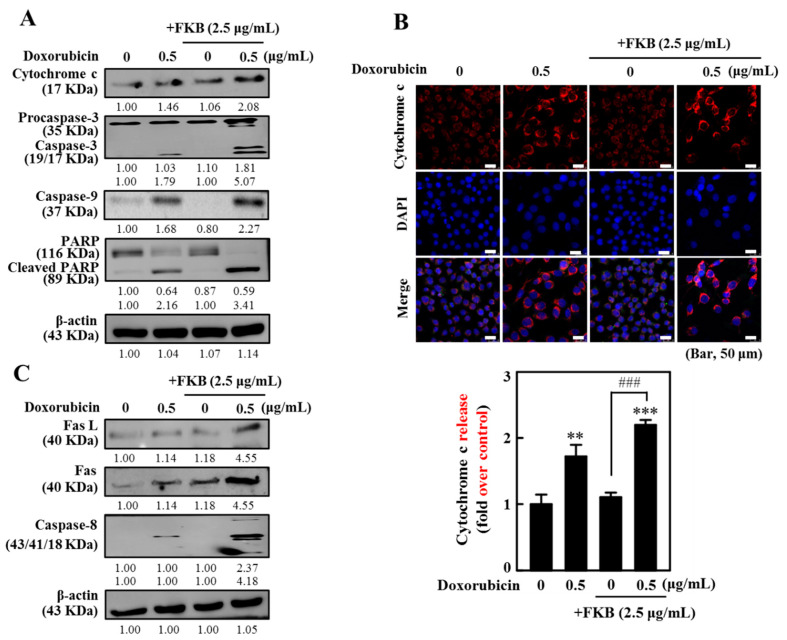
Induced apoptosis in AGS cells via the mitochondrial and death receptor pathways. The cells have been exposed to FKB (2.5 μg/mL), doxorubicin (0.5 μg/mL), and their 24-h combination. (**A**) Western blot analysis of the cytochrome levels c, caspase-3, caspase-9, and PARP (Figure S2). (**B**) Immunofluorescence staining indicates changes in expression of the cytochrome c. (**C**) Western blot analysis of the protein levels FasL, Fas, and caspase-8. It shows typical findings from three independent experiments. On SDS-PAGE, the protein (50 μg) was solved from each sample, and Western blot was performed. β-actin was used as an internal control of the loading. Densitometric analysis was used to measure relative changes in protein bands with the control as 1.0-fold, as shown just below the gel results (Figure S3). Values were expressed as mean ± SD (*n* = 3). Significant at ** *p* < 0.01; *** *p* < 0.001 compared to untreated control cells. Significant at ^###^ *p* < 0.001 compared to doxorubicin-treated cells.

To further delineate the manner in which doxorubicin and FKB induced apoptosis in AGS cells, mitochondrial-dependent apoptotic protein markers have been looked at using Western blotting analyses. Cytochrome c levels from the total fraction were assessed. The results of this study show that the combination of FKB and doxorubicin treatment greatly enhanced the cytochrome c in AGS cells (Figure 2A and Figure S2A). The cytochrome c release was also consistent with immunofluorescence image data (Figure 2B). In addition, the results showed that pro-caspase-3, which were highly expressed inactive forms in doxorubicin and FKB incubation alone, were significantly activated after the FKB+doxorubicin treatment (2.5 and 0.5 µg/mL, 24 h). An increase in caspase-9 was clearly observed in the FKB+doxorubicin treated cells (Figure 2A and Figure S2A). The effect of activated caspase-3 on PARP cleavage was established because the proteolytic cleavage of PARP by caspase-3 is a key characteristic of apoptosis. Cells incubated with FKB+doxorubicin developed in the proteolytic cleavage of 116 KDa PARP to an 89 KDa fragment (Figure 2A and Figure S2A). Both activated caspases and PARP by FKB+doxorubicin showed the induction of mitochondrial-dependent apoptotic cell death in AGS cells.

### 2.4. FKB Enhanced Doxorubicin-Mediated FasL/Fas Expression and Caspase-8 Activation in AGS Cells

Fas and Fas ligand (FasL) protein levels in AGS cells were determined by Western blotting to assess if the combination of FKB and doxorubicin promoted apoptosis via a death receptor-associated pathway. The results of this study indicated that doxorubicin stimulates the expression of Fas and FasL levels in AGS cells. However, a remarkable induction of Fas and FasL was observed with the combination treatment (Figure 2C and Figure S2C). In mitochondria-dependent pathways of apoptosis, caspase-8 proteolytically activates a Bid (pro-apoptotic protein). This targets mitochondrial membrane permeabilization and also represents a link between intrinsic and extrinsic apoptotic pathways [32]. Figure 2C shows that treatments with FKB+doxorubicin significantly increase the caspase-8 cleavages when compared with only doxorubicin as part of the treatment (Figure 2C and Figure S2C). Our results confirmed that the combination of FKB and doxorubicin was able to synergistically induce apoptosis by the mediated mitochondrial pathway as well as by death receptor signals via activation of the Fas-FasL system in AGS cells.

### 2.5. Caspase Inhibitor Attenuates the Induction of Apoptosis by FKB+Doxorubicin in AGS Cells 

**Figure 3:**
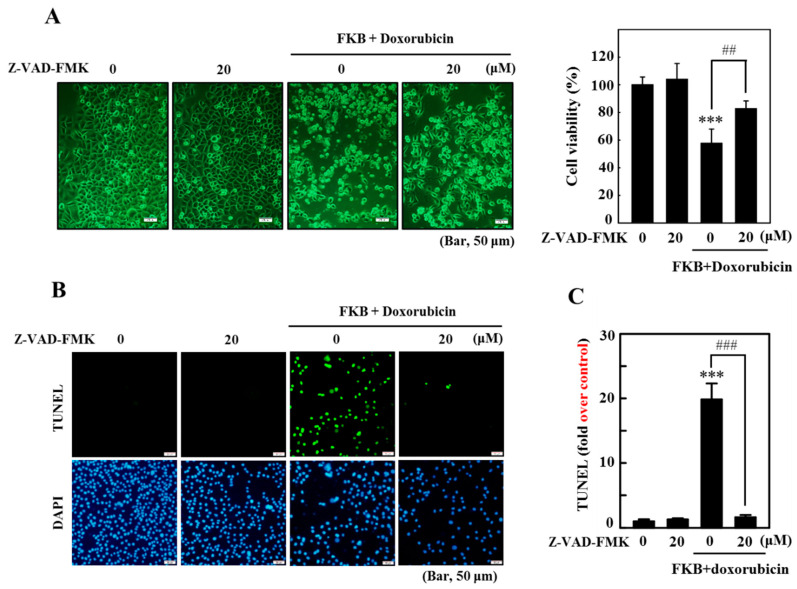
FKB exacerbates apoptotic cell death in AGS cells caused by doxorubicin. Cells were treated for 1 h with the apoptosis inhibitor (Z-VAD-FMK, 20 μM) prior to co-treatment with FKB (2.5 μg/mL) and doxorubicin (0.5 μg/mL) for 24 h. (**A**) Morphological changes were examined under a phase-contrast microscope and the MTT assay was performed on the viability of the cells. (**B**,**C**) A TUNEL assay was conducted to determine the fragmentation of apoptotic DNA. The green florescence shows the number of TUNEL-positive cells from three separate samples in the microscopic fields (Bar, 50 μM). Values were expressed as mean ± SD (*n* = 3). Significant at *** *p* < 0.001 compared to untreated control cells. Significant at ^##^ *p* < 0.01; ^###^ *p* < 0.001 compared to doxorubicin-treated cells.

To further understand whether FKB+Doxorubicin induced apoptotic cell death, we pretreated cells with an apoptotic inhibitor (Z-VAD-FMK) and determined the changes in the morphology, viability, and TUNEL assay. The Z-VAD-FMK pretreatment significantly attenuates FKB+doxorubicin-induced AGS cell death (Figure 3A). Further, AGS cells pre-treated with Z-VAD-FMK prior to FKB+doxorubicin resulted in a substantial inhibition of FKB+doxorubicin-induced apoptotic DNA fragmentation (Figure 3B,C), which confirms that the combination regimen induced apoptotic-mediated AGS cell death.

### 2.6. FKB+Doxorubicin Is Able to Activate Autophagy in AGS Cells from the Increased LC3 Accumulation

**Figure 4:**
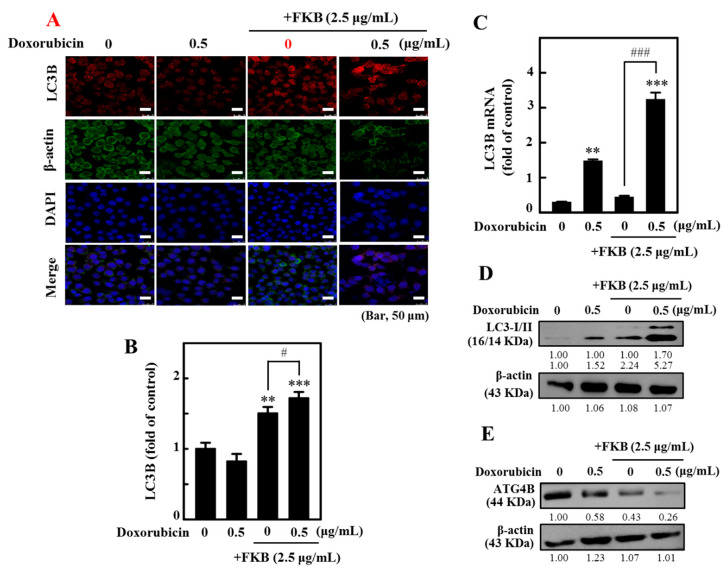
FKB increases the autophagy induced by doxorubicin in AGS cells. Cells have been exposed to FKB (2.5 μg/mL), doxorubicin (0.5 μg/mL), and a 24-h combination of these. (**A**,**B**) Staining with immunofluorescence indicates changes in the expression of LC3B. Using Olympus Softimage, the percentage age of fluorescence cell intensity of each experimental condition was quantified (**C**) RT-PCR analyses were used to determine the LC3B mRNA expression. (**D**) Western blot had determined the conversion of LC3-I to LC3-II (Figure S4A). (**E**) Western blot analysis of protein levels in ATG4B. Densitometric analysis measured relative changes in protein bands with the control as 1.0-fold as shown just below the gel data (Figure S4B). Values were expressed as mean ± SD (*n* = 3). Significant at ** *p* < 0.01; *** *p* < 0.001 compared to untreated control cells. Significant at ^#^ *p* < 0.05; ^###^ *p* < 0.001 compared to FKB-treated cells.

This study has speculated that FKB+doxorubicin might activate key regulatory proteins involved in autophagy. For this, we first examined the intracellular autophagy marker LC3 distribution in these cells [33] to determine if FKB+doxorubicin could induce autophagy in AGS cells. The results of this study indicated that the FKB+doxorubicin treatment significantly increased accumulations of lipidated LC3 form (LC3-II) when compared to the results of either FKB or doxorubicin alone (Figure 4A,B). The conversion of LC3-I to LC3-II upon FKB+doxorubicin incubation was convincingly increased in gene and protein levels (Figure 4C,D and Figure S4D). This study also demonstrated that the combination significantly inhibited the ATG4B expression in AGS cells when compared to the controls (Figure 4E and Figure S4E). 

### 2.7. AVOs in AGS Cells Are Enhanced by FKB+Doxorubicin Treatment

**Figure 5:**
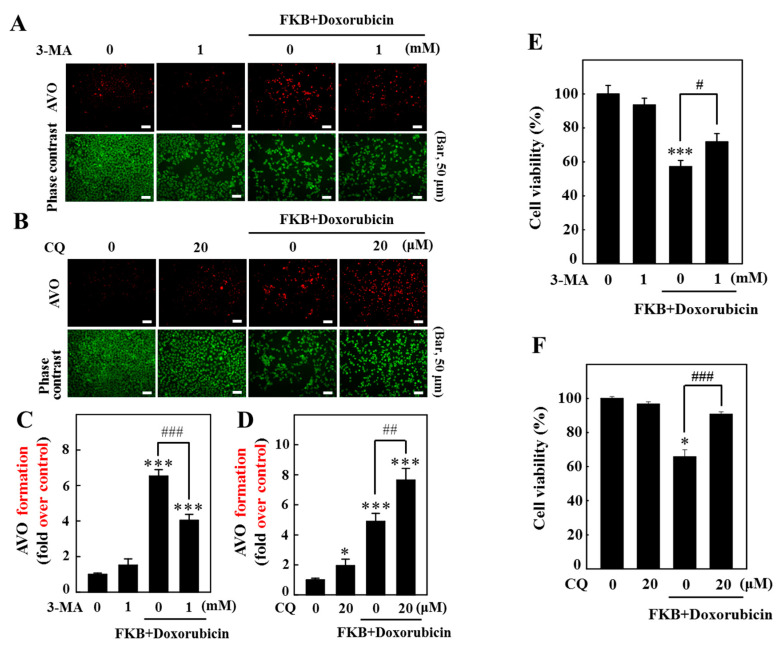
Pharmacologic autophagy inhibitors on FKB increase the formation of AVO triggered by doxorubicin in AGS cells. Cells were pretreated for 1 h with autophagy inhibitors (3-MA, 1 mM, and CQ, 20 μM), followed by a 24-h co-treatment with FKB (2.5 μg/mL) and doxorubicin (0.5 μg/mL). (**A**,**B**) The orange acridine (AO) was used to stain the AVOs. (**C**,**D**) Visualization of AVO formation in cells under a red filter fluorescence microscope (100× magnification). Red intensity of the fluorescence is proportional to the number of AVOs in cells. Number of stained AO cells was presented as histograms, with a control as 1.0-fold. (**E**,**F**) Autophagy triggered by FKB+doxorubicin which signaled cascades as a cell death mechanism. MTT trial determined cell viability. Values were expressed as mean ± SD (*n* = 3). Significant at * *p* < 0.05; *** *p* < 0.001 compared to untreated control cells. Significant at ^#^ *p* < 0.05; ^##^ *p* < 0.01; ^###^ *p* < 0.001 compared to FKB+doxorubicin-treated cells.

Autophagy is generally characterized by the formation of acidic vesicular organelles (AVOs). The effects of FKB+doxorubicin on AVO formation were detected under a fluorescence microscope using AVO staining. Figure 5A,C shows the pretreatment of cells with the pharmacological inhibitors 3-methyladenine (3-MA, inhibitor of early autophagy) and chloroquine (CQ, inhibitor of late autophagy). 3-MA effectively blocked FKB+doxorubicin-induced AVO formation. Interestingly, CQ pretreatment increased the expression of AVO formation under the FKB+doxorubicin treatment (Figure 5B,D). 

### 2.8. FKB+Doxorubicin-Treated AGS Cells Indicate the Activation of Autophagy Signaling Cascade as a Death Mechanism

The FKB+doxorubicin-induced autophagy’s role in AGS cells was examined by stopping the autophagy with 3-MA and CQ. Cells were treated with FKB+doxorubicin, 3-MA/CQ alone, or both to stop the autophagy. This study found that the cell pretreatment with 3-MA (1 mM) and CQ (20 µM) effectively enhances FKB+doxorubicin-induced cell death (Figure 5E,F). Our results show that the autophagy that is triggered by FKB+doxorubicin is also a death mechanism of death in AGS cells. 

### 2.9. FKB Enhanced Doxorubicin-Modulated Expression of Bcl-2 Family Proteins in AGS Cells 

**Figure 6:**
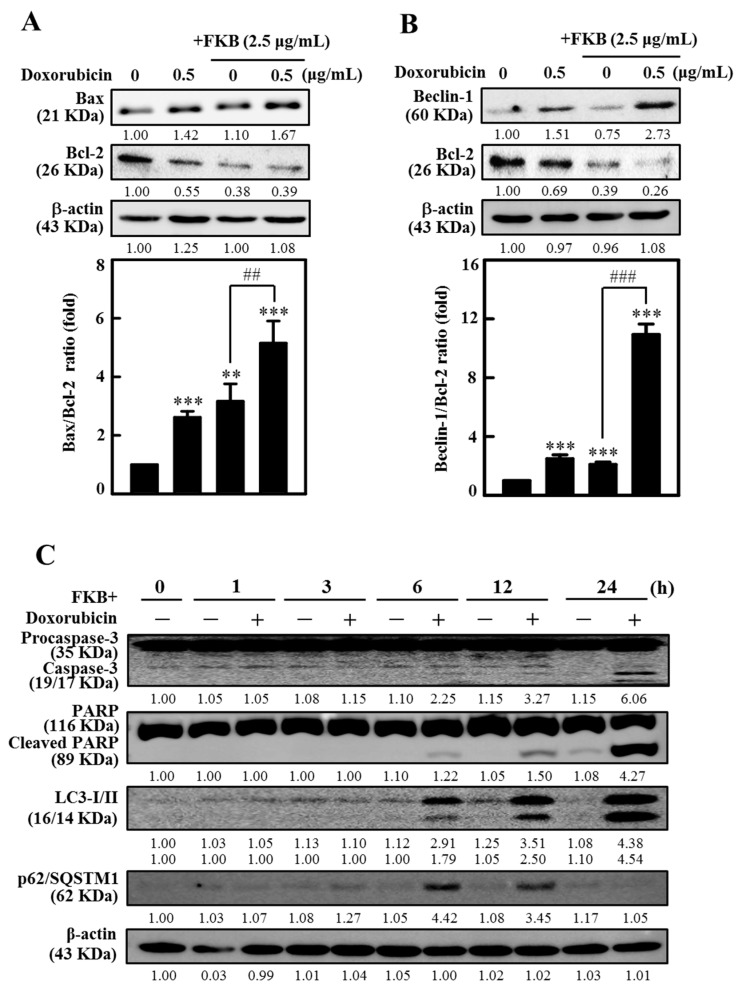
FKB+doxorubicin increased the ratio of AGS cells to Bax/Bcl-2 and Beclin-1/Bcl-2. Cells undergoing a 24-h co-treatment with FKB (2.5 μg/mL) and doxorubicin (0.5 μg/mL). (**A**) FKB+doxorubicin increases the ratio Bax/Bcl-2. Western blot determined changes in the proteins Bax and Bcl-2. Relative changes in the Bax/Bcl-2 ratio have been measured using commercially available quantitative software with a control representing 1-fold (Figure S5A). (**B**) The Beclin-1/Bcl-2 ratio increases with FKB+doxorubicin. Western blot determined changes in the Beclin-1 and Bcl-2 proteins expression. Relative changes in the Beclin-1/Bcl-2 ratio were measured by commercially available quantitative software with a control as 1-fold (Figure S5B). (**C**) Time-dependent changes in apoptotic proteins (caspase-3 and PARP) and autophagy markers (LC3-I/LC3-II and p62/SQSTM1), with co-treatment of 0, 1, 3, 6, 12, and 24 h after FKB (2.5 μg/mL) and doxorubicin (0.5 μg/mL) (Figure S5C). Values were expressed as mean ± SD (*n* = 3). Significant at ** *p* < 0.01; *** *p* < 0.001 compared to untreated control cells. Significant at ^##^ *p* < 0.01; ^###^ *p* < 0.001 compared to FKB+doxorubicin-treated cells.

The Bcl-2 family proteins play a key role in apoptosis mediated by mitochondria, either as activators (Bax, Bad, and Bok) or as inhibitors (Bcl-2, Bcl-xL, and Bcl-w) [34]. In AGS cells the effect of FKB enhanced doxorubicin on the levels of Bax and Bcl-2 expression was determined. Figure 6A and Figure S6A show that doxorubicin caused an increase in apoptotic levels of Bax protein and a dramatic reduction in anti-apoptotic levels of Bcl-2 protein compared to the control. Interestingly, the combination of FKB and doxorubicin significantly enhanced the Bax and Bcl-2 ratio as compared to doxorubicin alone (Figure 6A and Figure S6A). These results suggest that the combination of FKB and doxorubicin treatment may disturb the Bax/Bcl-2 ratio (Figure 6A and Figure S6A) and induce apoptosis in AGS cells. 

The interaction between anti-apoptotic protein, Bcl-2 and autophagy protein, Beclin-1 is complex. Bcl-2 reported to reduce the pro-autophagy property of Beclin-1, whereas Beclin-1 cannot neutralize the apoptotic function of Bcl-2 [11]. Based on this, we studied the effect of FKB+doxorubicin on the Bcl-2 protein and its role on Beclin-1 (pro-autophagic) expressions in AGS cells. Western blot data showed that Beclin-1 proteins were dramatically increased with the FKB+doxorubicin treatment (Figure 6B and Figure S6B). Consistent with this response, Bcl-2 expression was downregulated with the combination treatment (Figure 6B and Figure S6B). These results suggest that the combination of FKB and doxorubicin treatment may disturb the Beclin-1/Bcl-2 ratio (Figure 6B and Figure S6B) and induce autophagy in AGS cells. These results convinced that the lack of Bcl-2 in the presence of FKB+doxorubicin may tip the homeostasis in favor of apoptosis and autophagy in cancer cells. The quantified ratio of Bax/Bcl-2 and Beclin-1/Bcl-2 was disrupted by FKB+doxorubicin, which suggest the induction of apoptosis and autophagy. In addition, Western blot data also demonstrated that the combination treatment showed significant changes in the expressions of PARP, caspase-3, LC3-I/LC3-II, or p62/SQSTM1 proteins in a time-dependent fashion (Figure 6C and Figure S6C), confirming the initiation of apoptosis and autophagy by FKB+doxorubicin in AGS cells. 

### 2.10. Inhibition of Autophagy Suppressed FKB+Doxorubicin-Induced Apoptosis

**Figure 7:**
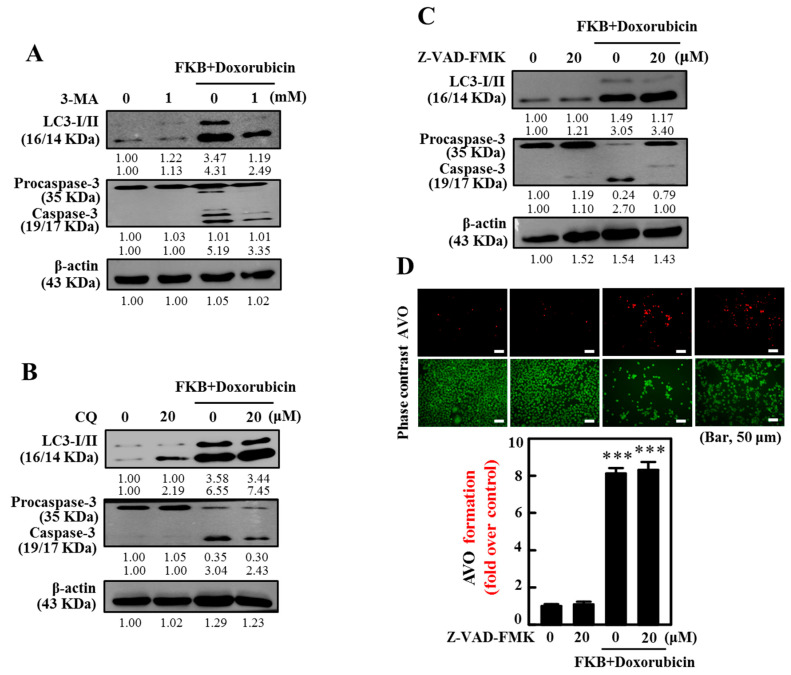
Interaction between the apoptosis induced by FKB/doxorubicin and AGS cell autophagy. (**A**,**B**) Inhibition of autophagy caused by FKB/doxorubicin suppressed apoptosis. Cells were pretreated with 3-MA (1 mM) (**A**, Figure S6A) or CQ (20 μM) (**B**, Figure S6B) autophagy inhibitors for 1 h, followed by a 24-h co-treatment with FKB (2.5 μg/mL) and doxorubicin (0.5 μg/mL). Western blot tracked shifts in the LC3-I/II and caspase-3 expressions. (**C**,**D**) Prevent autophagy inhibition of FKB-induced apoptosis. Cells were pretreated with Z-VAD-FMK (20 μM) caspase inhibitor for 1 h, followed by 24-h co-treatment with FKB (2.5 μg/mL) and doxorubicin (0.5 μg/mL). (**C**) Western blot monitored changes in the LC3-I/II and caspase-3 expressions (Figure S6C). (**D**) AVO formation in cells was visualized under the fluorescence microscope of the red filter (100× magnification). Red intensity of the fluorescence is proportional to the number of AVOs in cells. Number of stained AO cells was presented as histograms, with a control as 1.0-fold. Values were expressed as mean ± SD (*n* = 3). Significant at *** *p* < 0.001 compared to untreated control cells.

Next, we determined the autophagy flux by pretreatment of AGS cells with autophagy inhibitors 3-MA or CQ, which inhibits early or late autophagy. Treatment with autophagy inhibitors suppresses the LC3-II accumulation. These autophagy inhibitors were led by suppressed caspase-3 activation in AGS cells, which implies that inhibition of autophagy suppressed FKB+doxorubicin induced apoptosis (Figure 7A and Figure S7A; Figure 7B and Figure S7B). 

### 2.11. Inhibition of Apoptosis Suppressed FKB+Doxorubicin-Induced Autophagy

Normally caspases are in the inactive form, and their activation plays a crucial role in the execution-phase of apoptosis. On the other hand, the autophagy may proceed to activate the caspase-dependent apoptotic cell death under various circumstances [35]. To address whether there is an interaction between apoptosis and autophagy, we treated cells with the apoptosis inhibitor Z-VAD-FMK and changes in co-localization of LC3-I/LC3-II and caspase-3 activation were determined by Western blot and AVO formation. Results show that the inhibition of caspase-3 activation by Z-VAD-FMK resulted in weakened LC3-II accumulation and AVO formation (Figure 7C,D and Figure S7C), which suggests that the inhibition of apoptosis suppressed FKB+doxorubicin-induced autophagy. 

### 2.12. FKB+Doxorubicin Induced Apoptosis and Autophagy in AGS Cells Through JNK and ERK Signaling Pathways 

**Figure 8:**
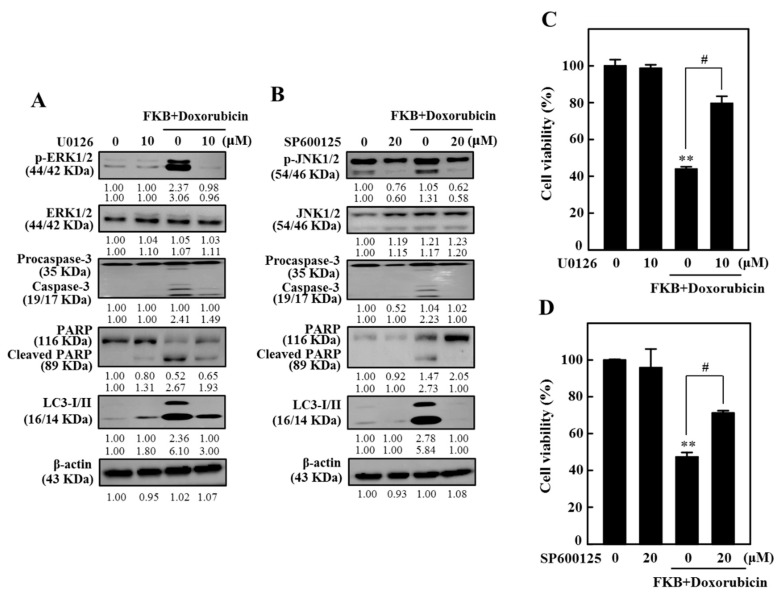
FKB+doxorubicin mediated apoptotic-and autophagic-cell death by upregulating pathways of ERK and JNK in AGS cells. Cells were pretreated for 30 min with U0126 (10 μM) or SP00125 (20 μM), followed by co-treatment with FKB (2.5 μg/mL) and doxorubicin (0.5 μg/mL) for 24 h. (**A**,**B**) The effect of FKB and doxorubicin co-treatment on expression of LC3-I/II, caspase-3, and PARP was investigated and then changes in total and phosphorylated levels of ERK and JNK were assessed by Western blot, with caspase-3 and PARP expressions (Figure S7A,B). (**C**,**D**) The MTT assay was used to assess the cell viability. Values were set to mean ± SD (*n* = 3). Compared with untreated control cells, significant at ** *p* < 0.01. Significant in ^#^ *p* < 0.05 compared to cells treated with FKB+doxorubicin.

FKB significantly enhanced the activation of p-ERK and p-JNK [27]. We subsequently determined the effect of ERK and JNK on cell viability, apoptosis, and autophagy with FKB and doxorubicin combination. Cells were pre-treated with MAPK inhibitors for ERK (U0126, 10 µM) or JNK (SP600125, 20 µM) for 30 min and then co-treated with FKB (2.5 µg/mL) and doxorubicin (0.5 µg/mL) combination for 24 h. Intriguingly, pretreatment of JNK and ERK inhibitors significantly attenuates FKB+doxorubicin-induced activation of caspase-3, PARP cleavage, and LC3-I/II expression (Figure 8A,B and Figure S8A,B). Furthermore, ERK and JNK inhibitors pretreatment significantly inhibited FKB+doxorubicin-induced cell death in AGS cells (Figure 8C,D). 

### 2.13. FKB+Doxorubicin Induces ROS-Mediated Apoptotic and Autophagic Cascades in AGS Cells

**Figure 9:**
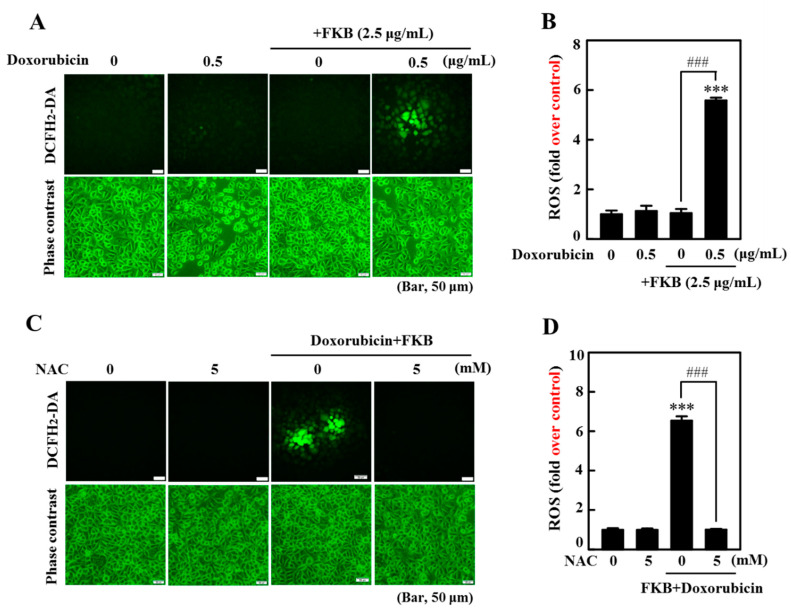
ROS generation in AGS cells was induced by FKB+doxorubicin. (**A**,**B**) FKB exacerbates intracellular ROS generation triggered with doxorubicin. The generation of ROS was measured at 60 min following co-treatment with FKB (2.5 μg/mL) and doxorubicin (0.5 μg/mL). (**C**,**D**) Antioxidant NAC prevents generation of FKBs and doxorubicin triggered ROS. Cells were pretreated with or without NAC (5 mM, 1 h) followed by exposure to FKB (2.5 μg/mL) and doxorubicin (0.5 μg/mL) co-treatment for the indicated time. The DCFH2-DA fluorescence method was used. The DCFH2-DA probe reacts with a cellular ROS and is metabolized into fluorescent DCF. The amount of fluorescent DCF is proportionate to ROS production. The ROS concentrations are depicted as control folds in the graph. Values were expressed as mean ± SD (*n* = 3). Significant at *** *p* < 0.001 compared to untreated control cells. Significant at ^###^ *p* < 0.001 compared to FKB- or FKB+doxorubicin-treated cells.

**Figure 10:**
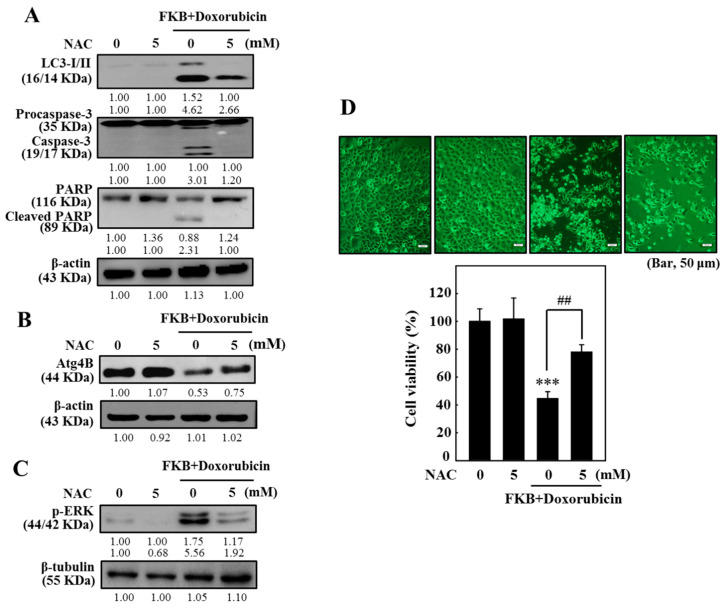
In AGS cells, ROS induced by FKB+doxorubicin is mediated-apoptosis and autophagy. Cells were pretreated for 1 h with an ROS inhibitor (NAC, 5 mM), and co-treated with FKB (2.5 μg/mL) and doxorubicin (0.5 μg/mL) for 24 h. (**A**) Western blot monitors the terms caspase-3, PARP, and LC3-I/II (Figure S8A). (**B**,**C**) Western blot was monitored for the terms ATG4B and p-ERK (Figure S8B,C). (**D**) The MTT assay was used to assess cell viability. Values were expressed as mean ± SD (*n* = 3). Significant at *** *p* < 0.001 compared to untreated control cells. Significant at ^##^ *p* < 0.01 compared to FKB-treated cells.

Excessive generation of ROS causes mitochondrial damage, which then triggers cellular autophagy and/or apoptosis [12,13,36]. To address the effects of FKB+doxorubicin on ROS generation, AGS cells were treated with FKB+doxorubicin for 60 min. We found that the increase in DCF fluorescence in FKB+doxorubicin-treated cells was directly proportionate to the amount of ROS (Figure 9A,B). In contrast, cells treated with the ROS inhibitor (NAC, 5 mM) 1 h prior to the FKB+doxorubicin treatment showed inhibition of ROS generation (Figure 9C,D). These results indicated that FKB+doxorubicin triggered ROS production in AGS cells, potentially propagating cell death. To further evaluate if FKB+doxorubicin-induced apoptosis and autophagy are ROS-dependent, AGS cells were pretreated with an ROS inhibitor (5 mM NAC, 1 h) followed by treatment with FKB+doxorubicin for the determination of apoptotic and autophagy markers. Western blot data showed that cells pretreated with NAC significantly downregulated the FKB+doxorubicin-induced LC-3 I/II expression, caspase-3 activation, and PARP cleavage (Figure 10A and Figure S10A). Similarly, FKB+doxorubicin-induced cell death was substantially blocked by the NAC pretreatment (Figure 10D). This shows that in human gastric carcinoma AGS cells, FKB+doxorubicin-induced apoptotic and autophagic cell death is mediated by ROS.

### 2.14. FKB+Doxorubicin Inhibits ROS-Related ATG4B and Enhances ERK Pathway in AGS Cells 

An initial study demonstrated that compared with the untreated control, the FKB+doxorubicin treatment significantly decreased the ATG4B expression in the AGS cells. The role of ROS in ATG4B-mediated activation of the autophagic pathway was determined using FKB+doxorubicin and ROS inhibitor (NAC). One of the notable observations is that the NAC pre-treatment substantially attenuated the FKB+doxorubicin-induced loss of the ATG4B levels (Figure 10B and Figure S10B). These results provide novel insights that ROS plays a critical role in the regulation of ATG4B levels in AGS cells. Further, the pre-treatment of AGS cells with NAC significantly weakened the FKB+doxorubicin-induced p-ERK expression indicating the role of ROS in the expression of ERK-induced apoptosis and autophagy signaling cascade (Figure 10C and Figure S10C).

### 2.15. Combination of Cisplatin and FKB-Induced Cell Death, Apoptosis, and Autophagy in AGS Cells

**Figure 11:**
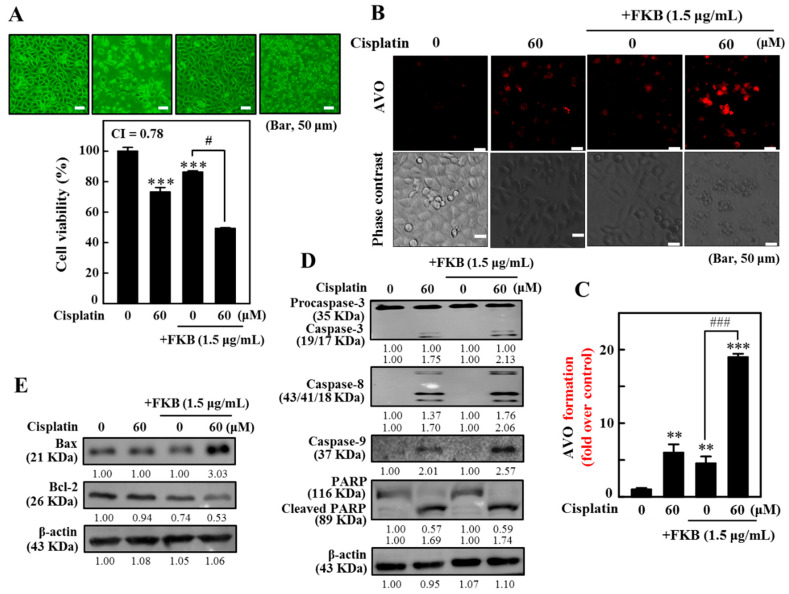
The FKB enhances/exacerbates/augments apoptosis and autophagy induced by cisplatin in AGS cells. Cells were treated for 24 h in combination with FKB (1.5 μg/mL), and/or cisplatin (60 μM). (**A**) Assessing cell viability using an MTT assay. According to the Chou and Talalay method, the effect of individual or co-treatments of both FKB and cisplatin on AGS cell survival and proliferation propagation were determined after the incubation period. The low value of the combination index (CI) signifies a greater synergistic effect. (**B**,**C**) The formation of AVOs were visualized through a fluorescent microscope (100× magnification, red filter). Quantification of AVO formation. (**D**) Western blot study of the expression caspase-3, caspase-8, caspase-9, and PARP (Figure S9A). (**E**) The co-treatment with FKB and cisplatin increases the ratio Bax/Bcl-2. Western blot determined changes in the expression of Bax and Bcl-2 proteins (Figure S9B). Relative changes in the Bax/Bcl-2 ratio were measured by comparing it with a control, whose value was arbitrarily considered as 1. Values were expressed as mean ± SD (*n* = 3). Significant at ** *p* < 0.01; *** *p* < 0.001 compared to untreated control cells. Significant at ^#^ *p* < 0.05; ^###^ *p* < 0.001 compared to FKB-treated cells.

Exposure to cisplatin resulted in significant growth inhibition, whereas the FKB+cisplatin co-treatment significantly inhibited the AGS cell line as compared to cisplatin alone (Figure 11A). Further, cells treated with the combinatorial regimen of FKB and cisplatin showed numerous progressive AVOs in the cytoplasm (Figure 11B,C). Western blotting analyses showed that the FKB+cisplatin combination regimen significantly induced caspase-3/-8/-9 activation and PARP cleavage as compared to FKB or cisplatin alone (Figure 11D and Figure S11D). In addition, FKB+cisplatin significantly alters the Bax/Bcl-2 ratio (Figure 11E and Figure S11E), which denotes that both apoptotic and autophagic cell death exists in AGS cells following FKB and cisplatin co-treatment.

### 2.16. In Vivo Tumor Growth Inhibition by FKB+Doxorubicin Treatment in Nude Mice 

**Figure 12:**
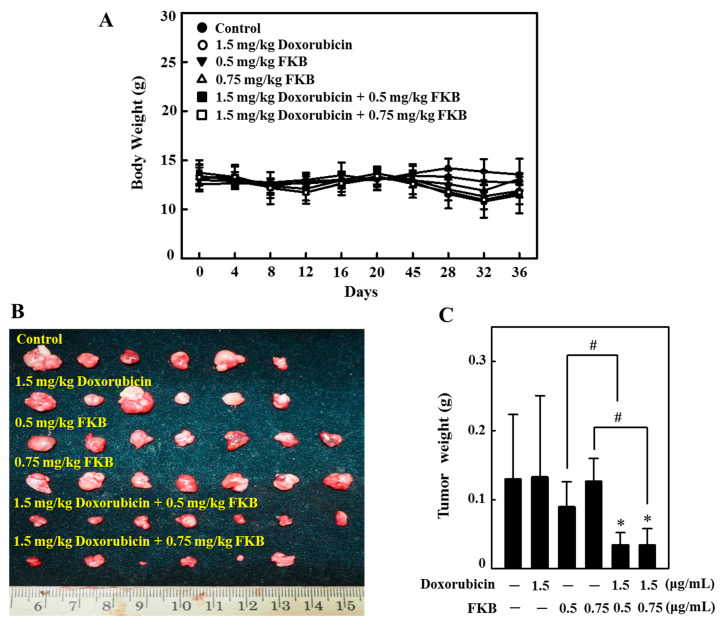
In vivo inhibition by co-treatment with FKB and doxorubicin of AGS-xenografted tumors in nude mice. BALB/c nude mice were intraperitonially injected with a vehicle (control) or doxorubicin (1.5 mg/kg, every two days) or FKB (0.5 mg or 0.75 mg/kg, every two days) or a combination of FKB and doxorubicin as indicated. (**A**) Body weight was measured 36 days every four days. (**B**,**C**) At the end of the 36th day animals were sacrificed, tumor tissues were removed, and weighed. Data were expressed as mean ± SD (*n* = 3). Significant at * *p* < 0.05 compared to untreated control cells. Significant at ^#^ *p* < 0.05 compared to FKB-treated cells.

Nude mice were used to investigate the in vivo effects of FKB, doxorubicin, and their combination effect on tumor growth. AGS cells were subcutaneously xenografted into nude mice. All animals appeared healthy, with no loss of body weight noted during the FKB treatment (Figure 12A). After 36 days, animals were sacrificed and an AGS-xenografted tumor was excised. Tumor volume data indicated that the combination treatment resulted in a more pronounced and significant inhibition of tumor growth as compared to the FKB and doxorubicin alone (Figure 12B,C). the analysis of our data strongly suggests that the combination regimen promoted anti-tumor activity in nude mice bearing AGS xenografts. 

## 3. Discussion

Increasing the dose of the anti-cancer drugs is necessary to overcome even a small increase in resistance to cancer cells that often leads to severe cytotoxicity off the target normal tissue. Strategies using dual inhibitors, rather than working through separate molecular mechanisms, are therefore considered the most effective choices for achieving higher curability via cancer chemotherapy with less toxicity [37]. Doxorubicin has been widely used to treat a wide variety of cancers for decades, but its efficacy is often limited by the development of resistance and cytotoxicity. Cardiac toxicity in cancer treatment with doxorubicin demands attention. Newer approaches involve the use of natural substance with an anti-cancer potential in conjunction with the anti-cancer drugs to achieve better efficacy for cancer chemotherapy. Flavokawain B (FKB) mediates several signaling pathways that exhibit inhibitory effects in breast cancer, osteosarcoma, bladder cancer, and synovial sarcoma. Therefore, our current research aims to increase the therapeutic efficacy of doxorubicin combined with FKB. We demonstrated that the FKB and doxorubicin treatment works synergistically to prevent gastric cancer cell proliferation by ROS-mediated apoptosis and autophagy pathways.

Mounting experimental evidence has confirmed that apoptosis can be triggered by (intrinsic) mitochondrial or (extrinsic) death receptor pathways. The mitochondrial/caspase-mediated signaling cascade is an important pathway of apoptosis characterized by mitochondrial outer membrane permeabilization (MOMP). The collapse of MOMP subsequently leads to the release of cytochrome c into the cytoplasm, triggering the caspase cascade and other apoptotic processes [38]. In this study, FKB+doxorubicin incubation activates cytochrome c, pro-caspase-9 and pro-caspase-3, and proteolytic cleavage of PARP, which subsequently leads to the induction of apoptotic cell death in AGS cells. A death receptor-mediated extrinsic pathway control is triggered by expression of FasL, Fas, and caspase-8, which sequentially promotes apoptotic cell death. Activation of caspase-8 triggers the proteolytic cleavage of mitochondria-associated Bid to induce apoptotic cell death following death receptor (FasL) activation [39]. Bid is a substrate for caspase-8 and a critical mediator for mitochondria-mediated apoptosis [40]. The present study indicates that FKB+doxorubicin treatment-induced apoptosis is associated with activation Fas, a cleavage of caspase-8 into active form in AGS cells. Taken together, our findings suggest that FKB+doxorubicin-induced apoptosis is controlled by both extrinsic and intrinsic pathways.

Autophagy, or self-eating, is a catabolic process of macromolecules and organelles degradation and recycling that serves various functions in gastric and solid tumor cancer. During autophagy, a cytosolic form of LC3 (LC3-I) is conjoined to phosphatidylethanolamine to form LC3-II by the activating enzyme ATG7 and ATG3 [41]. P62/SQSTM1 is an autophagy adaptor protein that functions as a signaling hub due to its ability to interact with signaling proteins [42]. A study has reported that the p62/SQSTM1 accumulation is required for resveratrol-induced autophagic cell death in cancer cells [43]. Consistent with previous reports, data obtained from the current study demonstrated that the FKB+doxorubicin co-treatment of AGS cells triggered the autophagy by increasing LC3-II accumulation, AVO formation, and p62/SQSTM1 expression levels. Concomitantly, the RT-PCR and Western blot results showed an upregulation of LC3 mRNA expression and protein levels. These findings suggest that the combination treatment promotes autophagy in AGS cells.

In anti-cancer therapies, apoptosis is a well-established cell death mechanism, whereas, the autophagy-mediated cell death mechanism is a fairly recent discovery. Autophagy is a type of caspase-independent cell death that does not involve DNA laddering, and is largely a result of intracellular autophagic degradation. Recent studies have shown increasing evidence that natural agents in several cancers can cause both apoptotic and autophagic cell death [35]. FKB+doxorubicin showed a strong cytotoxicity towards gastric cancer cells in humans; therefore, it is our interest to determine if this FKB+doxorubicin-mediated cell death is due to autophagy induction or apoptosis. A biochemical feature of apoptosis is considered to be a PARP-specific proteolytic cleavage by caspase-3. Interestingly, treatment with FKB+doxorubicin shows caspase-3 activation and PARP cleavage in AGS cells, whereas, pretreatment with Z-VAD-FMK significantly reduces the caspase-3, caspase-8, caspase-9, PARP cleavage, and Bax/Bcl-2 ratio of FKB+doxorubicin. LC3-I was converted to LC3-II through lipidation through ubiquitin-like conjugation during autophagosome formation [44]. The LC3-I to LC3-II expression ratio provides a simple measure of autophagy and was widely used as an autophagic marker. Current results indicate that FKB+doxorubicin induced AVO staining, and an increased protein expression ratio of LC3-II/LC3-I indicates that FKB+doxorubicin caused autophagy in AGS cells. In addition, FKB+doxorubicin-induced cell death was reversed by pretreatment with early (3-MA) or late (CQ) autophagy inhibitors. These results suggest that cell death caused by FKB+doxorubicin is mediated in AGS cells by autophagy and apoptosis.

The association between apoptotic (Bcl-2/Bcl-XL) and autophagy protein (Beclin-1) is a possible point of convergence of apoptotic and autophagic machinery [6,45]. Previous reports indicate that Beclin-1 will not neutralize the Bcl-2 anti-apoptotic activity, whereas Bcl-2 family proteins decrease the Beclin-1 pro-autophagic function [11]. In this study, the downregulated Bcl-2 and upregulated Beclin-1 treatment of FKB+doxorubicin in AGS cells indicate that FKB+doxorubicin activates both apoptosis and autophagy cascades, respectively. The activation of caspase-3 can cleave the Beclin-1 protein at 125 and 149 positions. The cleaved Beclin-1, which in turn disrupts its interaction with Bcl-2 then allows the release of pro-apoptotic molecules from the complex Bcl-2/Bcl-xL to initiate intrinsic apoptosis [46]. Moreover, the Beclin-1 caspase-mediated cleavage stimulates the crosstalk between apoptosis and autophagy [11]. Treatment with FKB+doxorubicin increased the ratio of Beclin-1/Bcl-2, and the ratio of Bax/Bcl-2 in AGS cancer cells indicates that FKB+doxorubicin can increase cell death through pro-apoptotic signaling.

Excessive production of ROS in cancer cells contributes to apoptosis or autophagy inducing and facilitating cell death [47]. The cells are able to maintain a state of redox homeostasis when oxidative stress is induced. Previous studies have shown that many stimuli, such as anti-cancer drugs, may induce cells to produce ROS by inducing the loss of the mitochondrial membrane potential [48,49]. Dysregulation of ROS levels and autophagy play an important role in the progression and initiation of cancer and have been recognized as potential cancer treatment targets [50]. Our present study showed that treatment with FKB+doxorubicin significantly induced fluorescence of DCF in AGS cells, indicating an increase in generation of intracellular ROS. Additionally, pretreatment with the NAC ROS inhibitor significantly reversed apoptosis induced by FKB+doxorubicin and accumulation of LC3-II. Based on these results, our findings suggest that in AGS cells ROS may be involved in the apoptosis/autophagy induced by FKB+doxorubicin.

ATG4B plays an important role in the ATG8/LC3 conjugation system, which is necessary for autophagosome formation. Decreased levels of ROS resulted in hyperactivation of cysteine endopeptidase ATG4B, leading to LC3 de-lipidation and defective autophagosome assembly [51]. Furthermore, the knockdown of the expression ATG4B has been associated with a decrease in cell viability and proliferation propagation of chronic myeloid leukemia cells leading to an accumulation of expression LC3-II and p62/SQSTM1 [52]. To corroborate such results, we studied the expression of the ATG4B protein in AGS cells by treating them with FKB+doxorubicin and NAC. Our data showed that treatment with FKB+doxorubicin activated the intracellular ROS generation, reduced ATG4B activity, and increased autophagic efficiency. The FKB+doxorubicin-enhanced intracellular ROS was attenuated due to the NAC pretreatment and has restored the ATG4B activity, which mitigated the autophagy induced by FKB+doxorubicin. Such observations indicate that FKB+doxorubicin can increase intracellular ROS, which contributes to ATG4B oxidation and inhibition, promotes LC3 lipidation (ATG8), and autophagy induction. Nevertheless, the extrapolation of the basic mechanism of action requires further investigation.

Mitogen-activated protein kinases (MAPKs), a family of serine/threonine kinases, including ERK and JNK play an important role in cell cycle regulation, apoptosis, and tumorigenesis [53]. ROS is involved in regulating the activation of the MAPK under different stress conditions. In our study, treatment with FKB+doxorubicin in gastric cancer cells leads to a significant change in the phosphorylated ERK and JNK levels. However, FKB+doxorubicin-induced apoptosis and autophagy were prevented by pretreatment of AGS cells with a JNK and ERK inhibitor. These results indicate that in human gastric carcinoma cells, through the activation of ERK and JNK signaling cascades FKB+doxorubicin activates autophagy and apoptosis.

## 4. Materials and Methods 

### 4.1. Chemicals 

GIBCO Laboratories (GIBCO BRL, Grand Island, NY, USA) collected RPMI 1640, Fetal bovine serum (FBS), penicillin-streptomycin (PS), and glutamine. Santa Cruz Biotechnology, Inc. (Heidelberg, Germany) has acquired antibodies against cytochrome c, Fas, FasL, Bcl-2, Bax, p-ERK1/2, ERK1/2, and β-actin. Cell Signaling Technology, Inc. (Danvers, MA, USA) procured antibodies against caspase-3, caspase-8, PARP, LC3-I/II, p62/SQSTM1, ATG4B, p-JNK1/2, JNK1/2, and Bcelin-1. Thermo Fisher Medical, Inc. (Waltham, MA, USA) has acquired an antibody against caspase-9. Santa Cruz Biotechnology (Santa Cruz, CA, USA) obtained all of the secondary antibodies. Calbiochem (San Diego, CA, USA) has obtained Z-Val-Ala-Asp-Fluoromethylketone (Z-VAD-FMK). Sigma-Aldrich (St. Louis, MO, USA) was used to remove 3-(4,5-dimethylthiazol-2-yl)-2,5-diphenyltetrazolium bromide (MTT), acridine black, 3-methyladenine (3-MA), or chloroquine (CQ), N-acetylcysteine (NAC), and 2′,7′-dihydrofluorescein-dictate (DCFH2-DA). Purchased from Pierce (Rockford, IL, USA) was the Enhanced Chemiluminescence (ECL) kit. All other chemicals were of the highest commercially available grade and were either supplied by Merck or Sigma Chemical Co. (St. Louis, MO, USA).

### 4.2. Drug Treatment 

As mentioned in our previous publication [24], FKB (HPLC grade, > 99% purity) was obtained from LKT Laboratories., Inc. (St. Paul, MN, USA). A stock solution of FKB (10 mg/mL or 35.2 mM) was prepared in 100% DMSO and then diluted in a medium. The final concentration of DMSO in the medium was 0.1%.

### 4.3. Cell Culture 

Cells from the American Type Culture Set (Rockville, MD, USA) were collected for human gastric cancer (AGS, SCM-1, and MKN45). These cells were grown in a humidified incubator in RPMI1640, supplemented with 10% FBS, 2 mM glutamine, 1% penicillin-streptomycin-neomycin (5% CO_2_ in air at 37 °C). Prior to the addition of FKB, cells were seeded onto 24-well plates. Cultures were harvested by counting cell suspensions with a hemocytometer and monitored for cell size.

### 4.4. MTT Assay 

Cells were seeded at a density of 5 × 10^4^ cells/well in 24-well plates. The cells were treated for 24 h with various concentrations of incubated FKB and doxorubicin. The cells were washed with PBS after incubation and subsequently incubated with 0.5 mg/mL MTT at 400 μL in a medium for 2 h. The culture supernatant was removed and resuspended with 400 μL of DMSO to dissolve the MTT formazan. Using an ELISA microplate reader (Bio-Tek Instruments, Winooski, VT, USA), the absorbance was measured at 570 nm. The effect of FKB and doxorubicin on cell viability was assessed as the percentage of viable cells compared to the control cells treated by the vehicle that were arbitrarily assigned 100% viability.

### 4.5. Drug Interaction Effects

To determine the combined effect of FKB and doxorubicin, AGS cells were treated with FKB and doxorubicin alone and for 24 h in combination with different doses, and growth inhibition was assessed using trypan blue exclusion. To assess the interaction, additivity, or antagonism of the two elements, the Chou et al. median-effect analysis and the Chou and Talalay combination index (CI) test were used [30]. Data are represented as a combination index (CI), where the synergistic, additive, and antagonistic effects of CI < 1, CI = 1, and CI > 1 are indicated. The CI values were determined on the basis of the following equation: α (D)1/(D x)1 + (D)2/(Dx) 2 + (D)1(D) 2/(D x) 1(D x) 2, where (Dx)1 and (Dx)2 are the x% inhibition doses for drug 1 and drug 2, respectively. The values predicted were determined as: (%A × %B)/100; and the CI index was measured using the CalcuSyn Biosoft (Biosoft, Cambridge, UK) software.

### 4.6. TUNEL Assay for Apoptotic DNA Fragmentation

As previously described, DNA fragmentation was calculated using the commercially available deoxynucleotidyl transferase-mediated dUTP-fluorescein nick end-labeling (TUNEL) assay kit (FragELTM DNA fragmentation detection kit, Calbiochem, San Diego, CA, USA) [54]. Briefly, apoptotic cells (2 × 10^4^ cells/mL in 8-well) were harvested after treatment with FKB and/or doxorubicin, fixed with 4% formaldehyde and mounted on glass slides. Fixed cells in TBS were permeabilized with 20 g/mL of proteinase K, and endogenous peroxidase in methanol was inactivated using 3% H_2_O_2_. The labeling of the 3′-OH ends of fragmented DNA with biotin-dNTP, using klenow at 37 °C for 1.5 h, observed apoptosis. The slides were then incubated with streptavidin conjoined with horseradish peroxidase, accompanied by incubation with 3,3′-diaminobenzidine and H_2_O_2_. Beneath a fluorescence microscope, their fluorescence nuclei identified the fragmented DNA. Under each setting, the fluorescence intensity has been quantified using a square segment of fluorescence-stained cells with an analySIS LS 5.0 soft image solution (Olympus Imaging America Inc., Corporate Parkway Center Valley, PA, USA), and the percentage of fluorescence intensity is directly proportional to the percentage of apoptotic cells, relative to that of untreated control cells.

### 4.7. Western Blotting

Cells (1 × 10^6^ cells/dish) were pretreated with FKB+doxorubicin in a 10-cm dish. Cells were then washed with cold PBS and re-suspended in a 10 mM Tris-HCl [pH 8.0] lysis buffer, 0.32 M sucrose, 1% Triton X-100, 5 mM EDTA, 2 mM DTT, and 1 mM phenylmethyl sulfonyl fluoride. At 4 °C the suspension was centrifuged for 20 min at 12,000 g. The total protein content was determined with the Bio-Rad assay reagent (Hercules, CA, USA). Proteins were separated by electrophoresis of the SDS-polyacrylamide gel (SDS-PAGE) and transferred to PVDF membranes afterwards. The blots were blocked at room temperature for 1 h with 5% non-fat milk in PBS containing 1% Tween-20 and incubated overnight with an appropriate primary antibody at 4 °C. On the following day, blots were incubated with a secondary antibody conjugated with peroxidase. The blots were imaged using an ImageQuantTM LAS 4000 mini (Fujifilm) system using a chemiluminescence substrate from SuperSignal West Pico (Thermo Scientific Inc., Rockford, IL, USA).

### 4.8. LC3 Immunofluorescence

Cells (10^4^ cells/well) were grown in an 8-well Lab-Tek chamber (Thermo Fisher Science, Waltham, MA, USA) in RPMI with 10% FBS. The cells had been pretreated with doxorubicin and FKB. The cells have been fixed for 15 min in 2% paraformaldehyde and permeabilized for 10 min with 0.1% Triton X-100. The cells were washed and blocked in PBS with 10% FBS, then incubated in 1.5% FBS with a primary antibody LC3B for 2 h. In 6% BSA, a secondary FITC (488 nm) antibody was incubated for another 1 h. The cell nuclei had been stained for 5 min with a solution of 1 μg/mL DAPI. The stained cells were washed with PBS and visualized at a 200 level magnification using a fluorescence microscope.

### 4.9. RNA Extraction and RT-PCR

We seeded cells at 5 × 10^5^ cells/dish in a 6-cm dish. The cells were treated with FKB and doxorubicin for 24 h after 90% confluence was reached. To extract RNA from the cultured cells, we used the TRIzol reagent (Invitrogen, NY, USA). For a reverse transcription polymerase chain reaction (RT-PCR) (Bio-Rad), 1 g of total sample RNA was used, with an amplification of 45 s at 94 °C for 30–38 cycles, annealing of 45 s at 65 °C, and a final extension of 1 min at 72 °C. LC3B F: 5′-TTACCTTCCCGAACATCGAC-3′ and LC3B R: 5′-GCATAATTCCACTGCAC-3′ were the primers used. Gel electrophoresis was used to validate PCR products in an agarose gel of 1%.

### 4.10. Acridine Orange Staining

The staining of acridine orange (AO) was used to detect AVOs forming in AGS cells. Cells were washed with PBS twice after the FKB+doxorubicin treatment, followed by AO staining (1 μg/mL) and PBS dilution containing 5% FBS for 15 min. Cells were washed with PBS after staining and coated with 5% FBS PBS. The cells were studied under a red filter fluorescence microscope, and flow cytometry visualized and analyzed the formation of AVOs in cells at a 100-point magnification. AO is a lysosomotropic, metachromatic, and a membrane-permeable fluorescent dye with a concentration-dependent fluorescent emission ranging from red (at high concentrations in lysosomes) to green (at low concentrations in cytosol), with yellow as an intermediate concentration under some conditions.

### 4.11. ROS Generation Assay

Fluorescence microscopy using DCFH2-DA has observed the intracellular aggregation of ROS. Cells (5 × 10^4^ cells/24-well) were grown in RPMI1640 supplemented with 10% FBS. Culture media was changed when cells reached 80% confluence. After the FKB+doxorubicin treatment, PBS washed cells were incubated with 10 μM DCFH2-DA present in the fresh culture medium. An intracellular esterase had eliminated the acetate groups on DCFH2-DA, trapping the probe within AGS cells. Intracellular ROS was measured with a fluorescence microscope as shown by DCF fluorescence (Olympus 1 al. 71 at a magnification of 200). Under each setting, the fluorescence intensity was quantified from a square section of fluorescence-stained cells using LS 5.0 soft imaging solutions analysis (Olympus Imaging America Inc., Corporate Parkway Centre Valley, PA, USA).

### 4.12. Animal Study

Female athymic nude mice (BALB/c-nu), 5–7 weeks of age, were purchased from the National Laboratory Animal Center (Taipei, Taiwan) and kept in caged housing at a specifically designed, pathogen-free, 12 h/12 h light/dark cycle insulation facility. All mice had free access to the rodent chow and water (Oriental Yeast Co Ltd., Tokyo, Japan). The animal studies were strictly enforced by the Chinese Society of Animal Science, Taiwan, publishing “The Instructions for the Care and Use of Laboratory Animals.” The animal protocols were accepted by the China Medical University’s Institutional Animal Care and Use Committee.

### 4.13. Tumor Cell Inoculation

A total of twenty-seven mice (5–7 weeks old) were randomly divided into nine groups containing three animals per group. AGS cells (5 × 10^6^ cells) were mixed with a 200 L matrix gel and subcutaneously injected into the left hind flanks of the nude mice. Seven days after inoculation, these mice were divided into the following groups: Control (0.1% DMSO), intraperitoneal injection of doxorubicin (1.5 mg/kg, every two days), FKB (0.5 mg/kg, every two days), FKB (0.75 mg/kg, every two days), doxorubicin+FKB (1.5 + 0.5 mg/kg, every two days), and doxorubicin+FKB (1.5 + 0.75 mg/kg, every two days). The body weight of each animal was assessed every four days to track the toxicity of the drugs. The tumor volume in mice was compared with caliper measurements of tumor volumes (length, width, and depth) that were measured every four days using the formula: Length × width^2^ × ½. Subsequently, all mice were sacrificed, tumor tissues (lung, spleen, liver, heart, and kidney) were removed and weighed. These tissues were examined by a veterinary pathologist.

### 4.14. Statistical Analyses

The data obtained were analyzed using variance analysis (ANOVA), followed by a pair wise comparison test by Dunnett. Mean values are shown with their standard deviation (in vitro, mean ± SD) or default (in vivo, mean ± SEM). Statistical significance for all tests between the treatments was established as *p* < 0.05.

## 5. Conclusions

Our results explain the cytotoxic effect of FKB and doxorubicin in a combination that is comparable to the high dose of groups treated with doxorubicin alone. Previous studies have shown that the chronic high-dose doxorubicin treatment induces toxicity associated with the drug. All in all, our data suggest that FKB helps reduce the requirement for the dose of doxorubicin, which contributes to a restriction of toxicity in cancer chemotherapy.

## Figures and Tables

**Table 1:** The synergism effects of FKB and doxorubicin on AGS cells.

Treatment	(μg/mL)	Cell Number (%)	Predicted Value ^#^	Combination Index (CI) ^##^
FKB	1.25	91.2 ± 2.0 **	—	—
	2.5	86.9 ± 4.2 **	—	—
	5	64.4 ± 1.2 ***	—	—
Doxorubicin	0.5	77.1 ± 3.7 ***	—	—
FKB + doxorubicin	1.25 + 0.5	66.4 ± 0.4 ***	70.3	0.64
	2.5 + 0.5	41.1 ± 2.6 ***	66.9	0.28
	5 + 0.5	6.8 ± 0.8 ***	49.6	0.07

^#^ Predicted value: (%A × %B)/100; ^##^ combination index according to Chou and Talalay [1], values < 1 indicates synergism; values are expressed as mean ± SD (*n* = 3). Significant at ** *p* < 0.01; *** *p* < 0.001 compared to untreated control cells.

**Table 2:** The synergism effects of FKB and doxorubicin on SCM-1 cells.

Treatment	(μg/mL)	Cell Number (%)	Predicted Value ^#^	Combination Index (CI) ^##^
FKB	1.25	98.9 ± 3.9	—	—
	2.5	96.3 ± 5.8	—	—
	5	76.8 ± 6.4 **	—	—
Doxorubicin	0.5	81.0 ± 1.1 ***	—	—
FKB + doxorubicin	1.25 + 0.5	61.4 ± 3.8 ***	80.1	0.53
	2.5 + 0.5	57.2 ± 1.6 ***	78.0	0.62
	5 + 0.5	43.6 ± 3.1 ***	62.2	0.67

^#^ Predicted value: (%A × %B)/100; ^##^ combination index according to Chou and Talalay [1], values < 1 indicates synergism; values are expressed as mean ± SD (*n* = 3). Significant at ** *p* < 0.01; *** *p* < 0.001 compared to untreated control cells.

**Table 3:** The synergism effects of FKB and doxorubicin on MKN45 cells.

Treatment	(μg/mL)	Cell Number (%)	Predicted Value ^#^	Combination Index (CI) ^##^
FKB	1.25	98.1 ± 7.0	—	—
	2.5	94.9 ± 4.7	—	—
	5	78.7 ± 3.7 **	—	—
Doxorubicin	0.5	87.7 ± 3.5 *	—	—
FKB + doxorubicin	1.25 + 0.5	65.0 ± 4.1 ***	86.0	0.57
	2.5 + 0.5	63.4 ± 2.2 ***	83.1	0.70
	5 + 0.5	41.4 ± 3.7 ***	68.9	0.59

^#^ Predicted value: (%A × %B)/100; ^##^ combination index according to Chou and Talalay [1], values < 1 indicates synergism; values are expressed as mean ± SD (*n* = 3). Significant at * *p* < 0.05; ** *p* < 0.01; *** *p* < 0.001 compared to untreated control cells.

## Supplementary Materials

The following are available online at https://www.mdpi.com/2072-6694/12/9/2475/s1. Figure S1. Uncropped Western blot images of the protein content of caspase-3, related to Figure 1C. Figure S2. Uncropped Western blot images of the cytochrome levels c, caspase-3, caspase-9, and PARP, related to Figure 2A. Figure S3. Uncropped Western blot images of the protein levels FasL, Fas, and caspase-8, related to Figure 2C. Figure S4. (A) Uncropped Western blot images of the conversion of LC3-I to LC3-II, related to Figure 4A. (B) Uncropped Western blot images of protein levels in ATG4B. Densitometric analysis measured relative changes in protein bands with the control as 1.0-fold as shown just below the gel data, related to Figure 4E. Figure S5. (A) Uncropped Western blot images of the proteins Bax and Bcl-2. Relative changes in the Bax/Bcl-2 ratio have been measured using commercially available quantitative software with a control representing 1-fold, related to Figure 6A. (B) Uncropped Western blot images of the Beclin-1 and Bcl-2 proteins expression. Relative changes in the Beclin-1/Bcl-2 ratio were measured by commercially available quantitative software with a control as 1-fold, related to Figure 6B. (C) Uncropped Western blot images of the apoptotic proteins (caspase-3 and PARP) and autophagy markers (LC3-I/LC3-II and p62/SQSTM1), with co-treatment of 0, 1, 3, 6, 12, and 24 h after FKB (2.5 μg/mL) and doxorubicin (0.5 μg/mL), related to Figure 6C. Figure S6. (A,B) Uncropped Western blot images of Inhibition of autophagy caused by FKB/doxorubicin suppressed apoptosis. Cells were pretreated with 3-MA (1 mM), related to Figure 7A,B. (C) Uncropped Western blot images of the monitored changes in the LC3-I/II and caspase-3 expressions, related to Figure 7C. Figure S7. (A,B) Uncropped Western blot images of The effect of FKB and doxorubicin co-treatment on expression of LC3-I/II, caspase-3, and PARP was investigated and then changes in total and phosphorylated levels of ERK and JNK were assessed by Western blot, with caspase-3 and PARP expressions, related to Figure 8A,B. Figure S8. (A) Uncropped Western blot images of the terms caspase-3, PARP, and LC3-I/II, related to Figure 10A. (B,C) Uncropped Western blot images of The monitored for the terms ATG4B and p-ERK, related to Figure 10B,C. Figure S9. (A) Uncropped Western blot images of the the expression caspase-3, caspase-8, caspase-9, and PARP related to Figure 11D. (B) Uncropped Western blot images of The changes in the expression of Bax and Bcl-2 proteins, related to Figure 11E.
