# Supplementary material for: Flavokawain B and Doxorubicin Work Synergistically to Impede the Propagation of Gastric Cancer Cells via ROS-Mediated Apoptosis and Autophagy Pathways
Source: Cancers (Basel). 2020 Sep 1;12(9):2475. doi: 10.3390/cancers12092475 (PMC7564097; doi:10.3390/cancers12092475)
Supplement: Supplementary file 1 [file cancers-12-02475-s001.pdf]

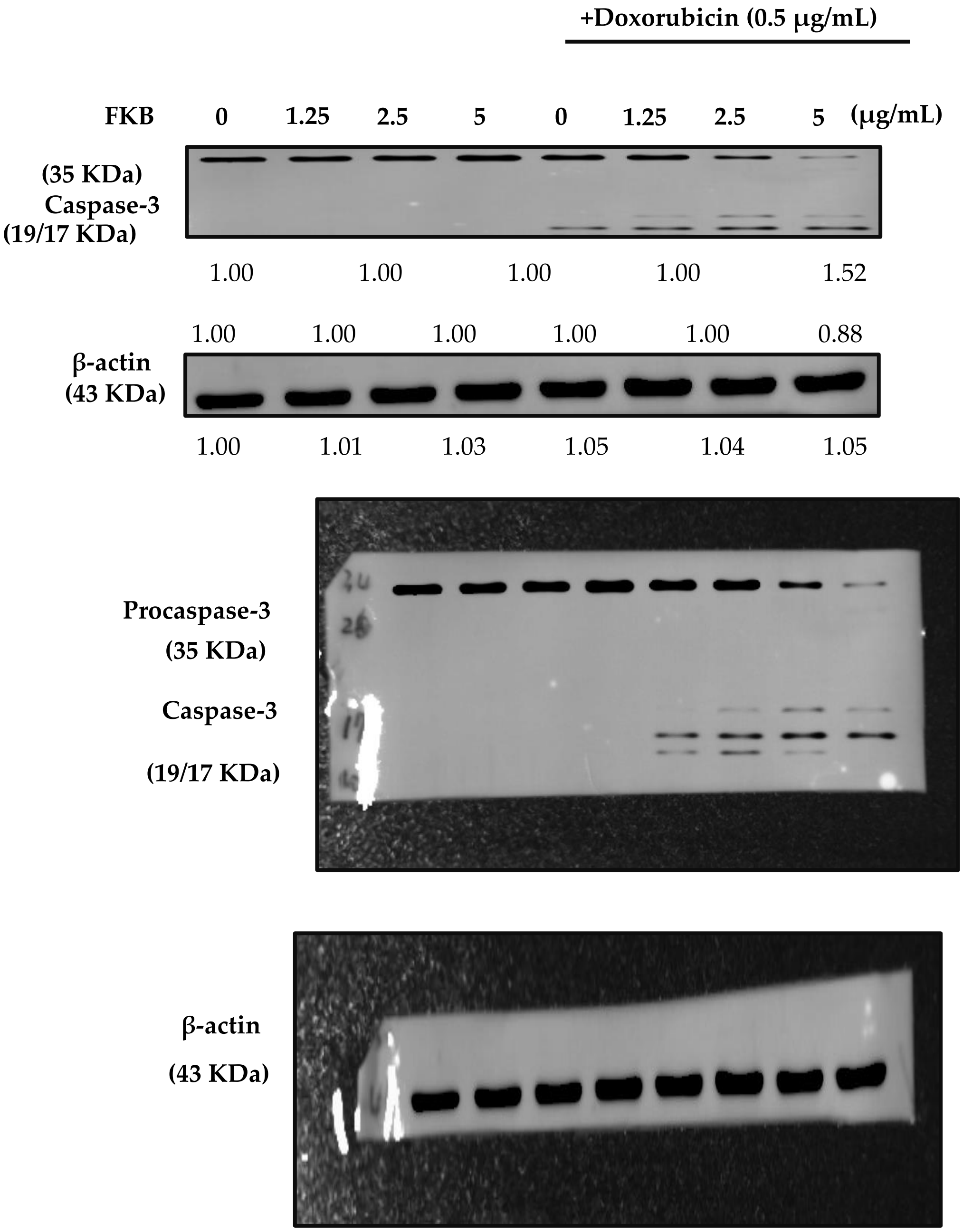

Figure S1. Uncropped Western blot images of the protein content of caspase-3, related to Figure 1C.

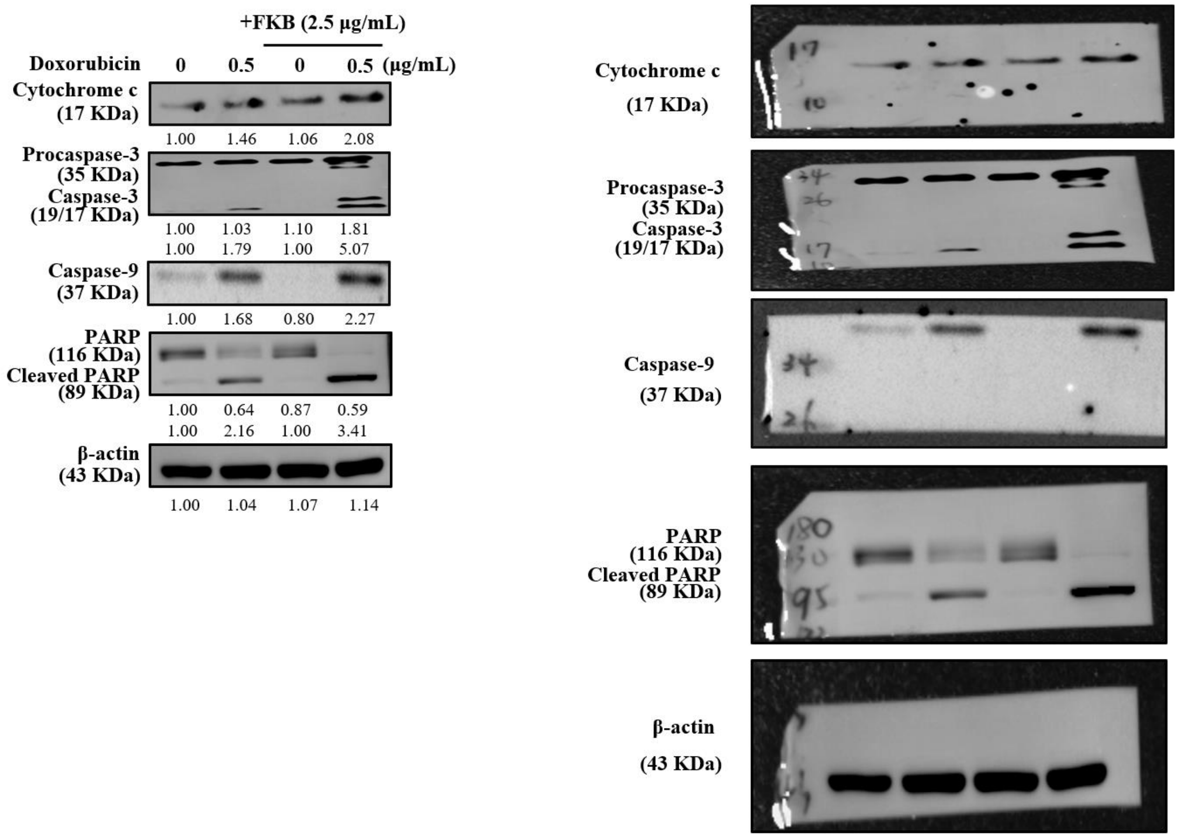

Figure S2. Uncropped Western blot images of the cytochrome levels c, caspase-3, caspase-9, and PARP, related to Figure 2A.

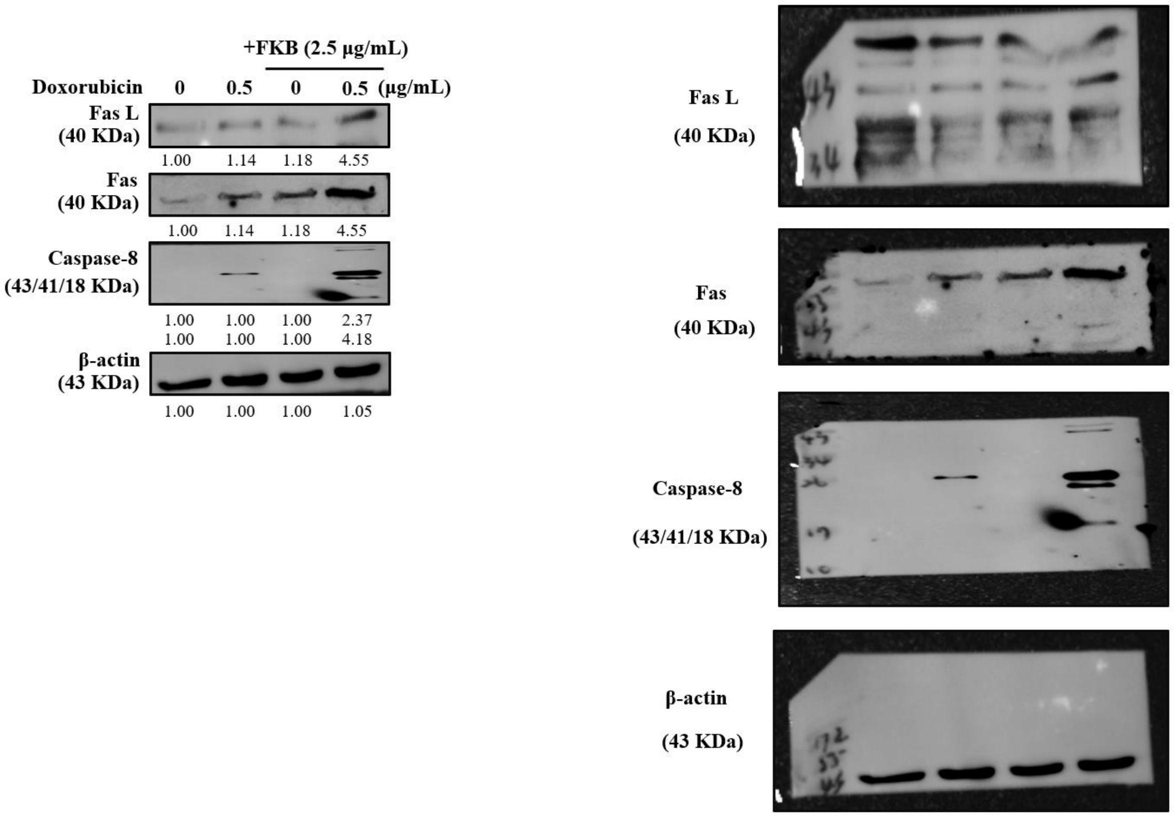

Figure S3. Uncropped Western blot images of the protein levels FasL, Fas, and caspase-8, related to Figure 2C.

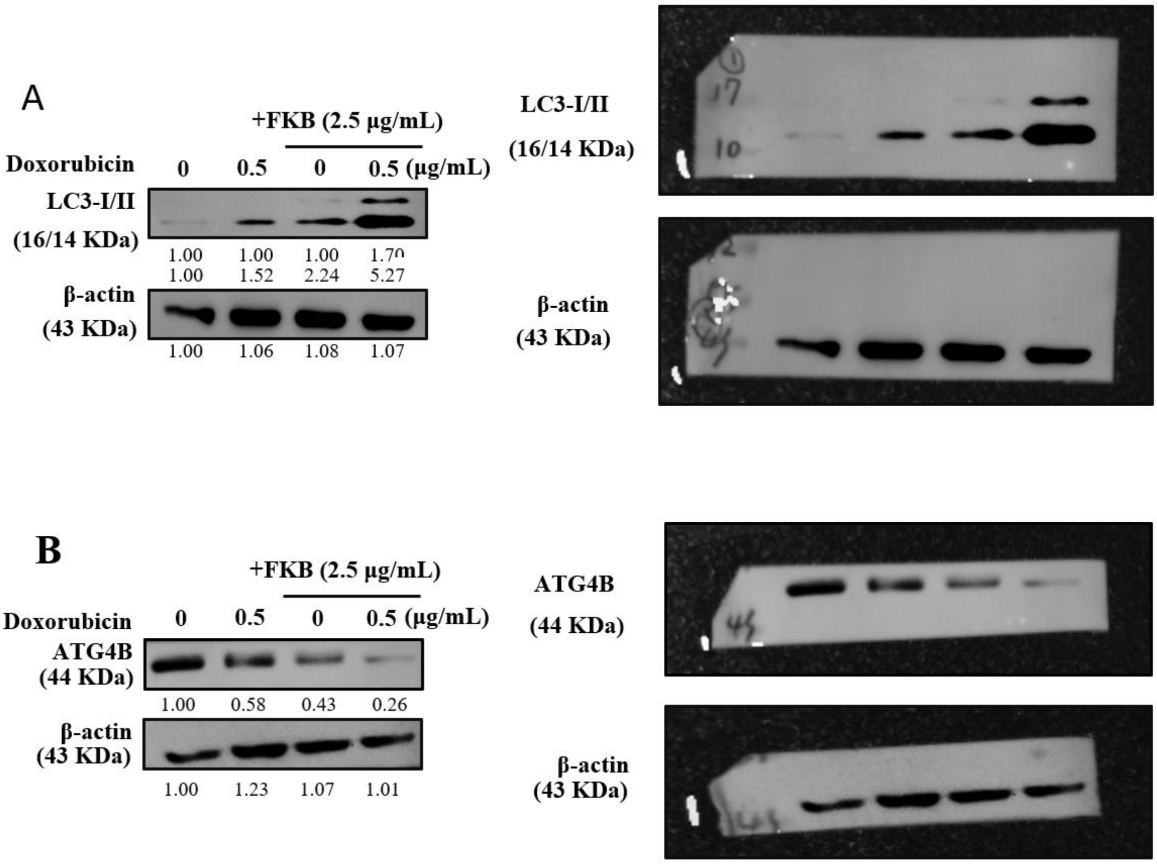

Figure S4. (**A**) Uncropped Western blot images of the conversion of LC3-I to LC3-II, related to Figure 4A. (**B**) Uncropped Western blot images of protein levels in ATG4B. Densitometric analysis measured relative changes in protein bands with the control as 1.0-fold as shown just below the gel data, related to Figure 4E.

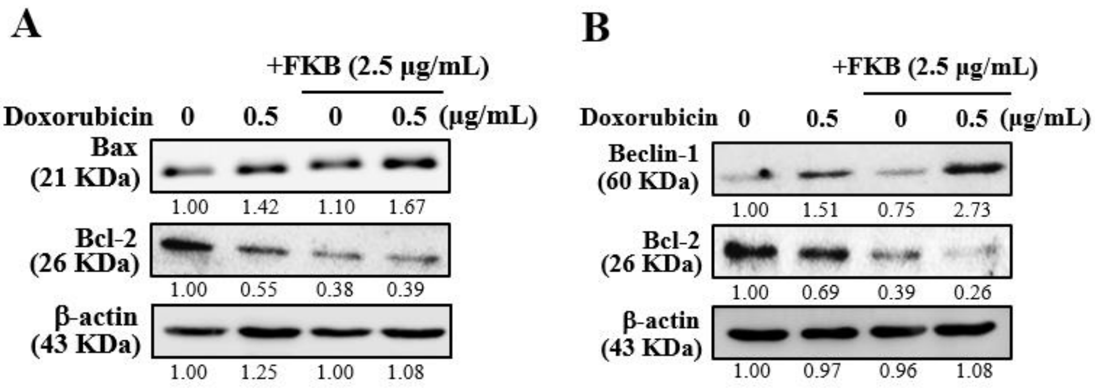
A
+FKB (2.5 μg/mL)
Doxorubicin 0 0.5 0 0.5 (μg/mL)
Bax (21 KDa)
1.00 1.42 1.10 1.67
Bcl-2 (26 KDa)
1.00 0.55 0.38 0.39
β-actin (43 KDa)
1.00 1.25 1.00 1.08
B
+FKB (2.5 μg/mL)
Doxorubicin 0 0.5 0 0.5 (μg/mL)
Beclin-1 (60 KDa)
1.00 1.51 0.75 2.73
Bcl-2 (26 KDa)
1.00 0.69 0.39 0.26
β-actin (43 KDa)
1.00 0.97 0.96 1.08

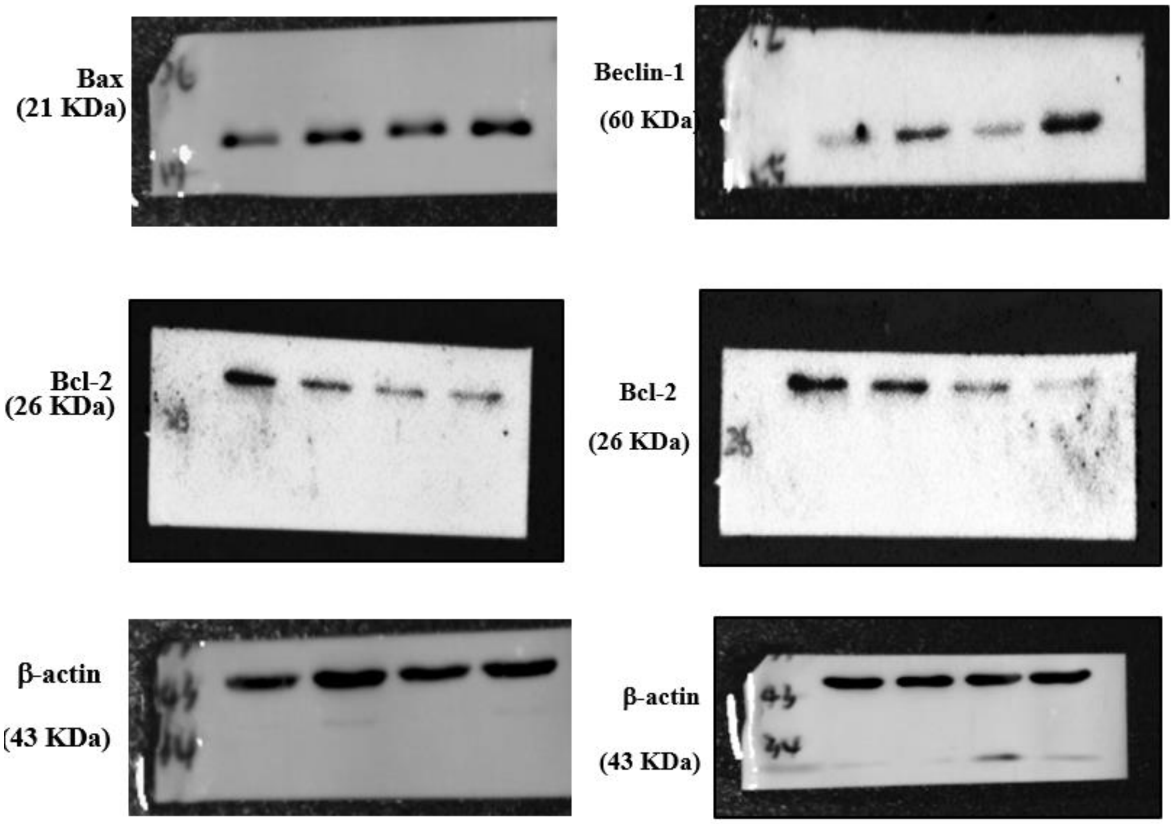
Bax (21 KDa)
Beclin-1 (60 KDa)
Bcl-2 (26 KDa)
Bcl-2 (26 KDa)
β-actin (43 KDa)
β-actin (43 KDa)

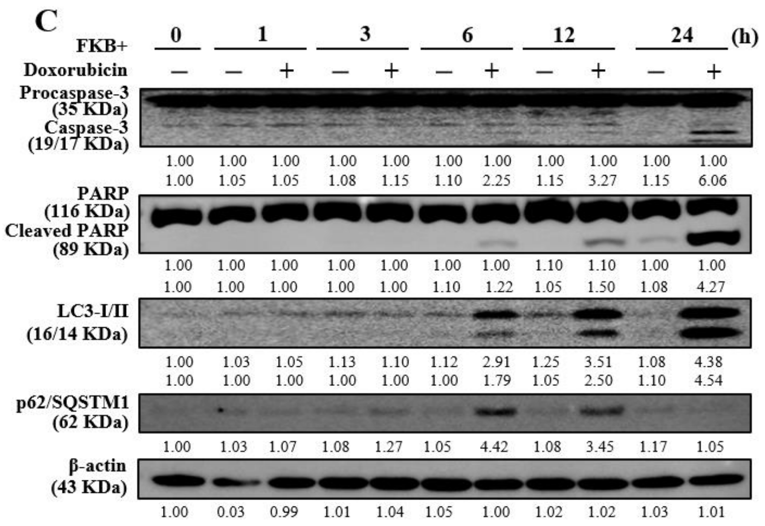

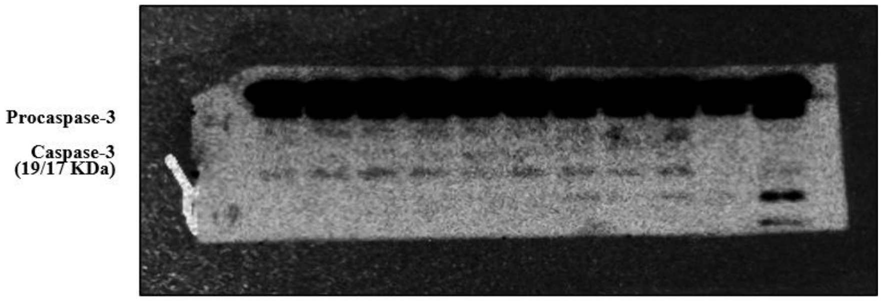

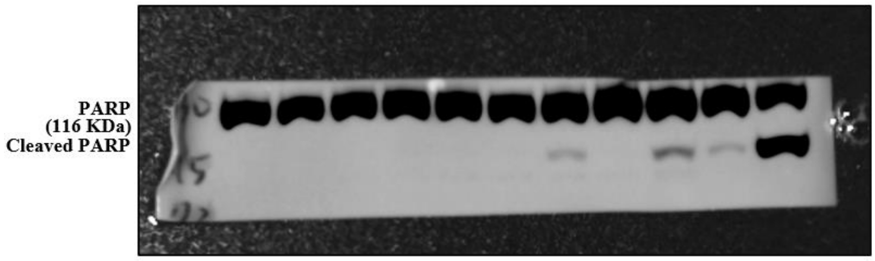

Figure S5. (**A**) Uncropped Western blot images of the proteins Bax and Bcl-2. Relative changes in the Bax/Bcl-2 ratio have been measured using commercially available quantitative software with a control representing 1-fold, related to Figure 6A. (**B**) Uncropped Western blot images of the Beclin-1 and Bcl-2 proteins expression. Relative changes in the Beclin-1/Bcl-2 ratio were measured by commercially available quantitative software with a control as 1-fold, related to Figure 6B. (**C**) Uncropped Western blot images of the apoptotic proteins (caspase-3 and PARP) and autophagy markers (LC3-I/LC3-II and p62/SQSTM1), with co-treatment of 0, 1, 3, 6, 12, and 24 h after FKB (2.5 μg/mL) and doxorubicin (0.5 μg/mL), related to Figure 6C.

A

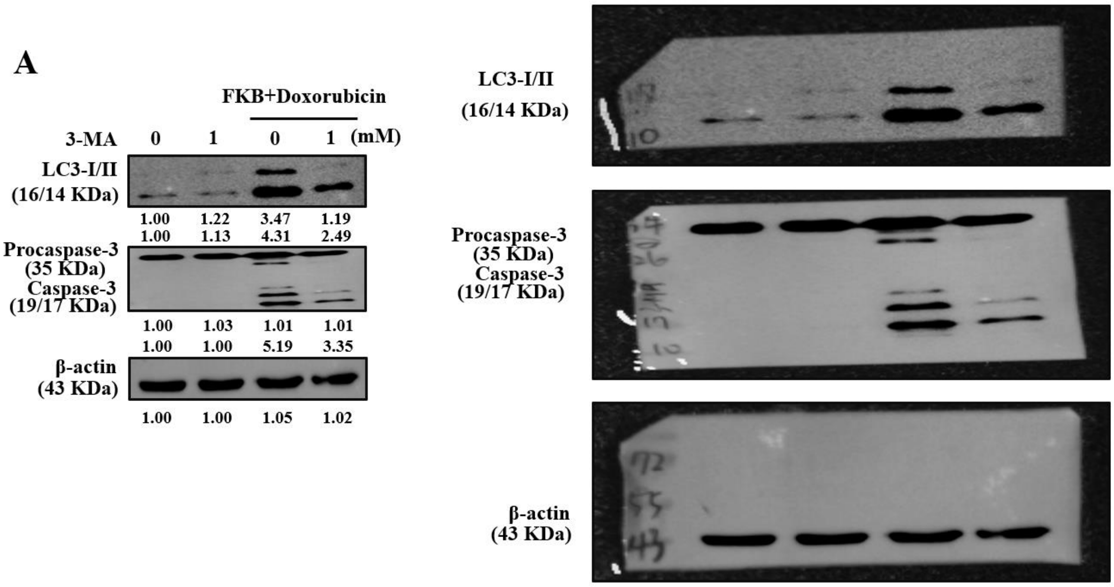
FKB+Doxorubicin
3-MA 0 1 0 1 (mM)
LC3-I/II
(16/14 KDa)
1.00 1.22 3.47 1.19
1.00 1.13 4.31 2.49
Procaspase-3
(35 KDa)
Caspase-3
(19/17 KDa)
1.00 1.03 1.01 1.01
1.00 1.00 5.19 3.35
β-actin
(43 KDa)
1.00 1.00 1.05 1.02
LC3-I/II
(16/14 KDa)
Procaspase-3
(35 KDa)
Caspase-3
(19/17 KDa)
β-actin
(43 KDa)

B

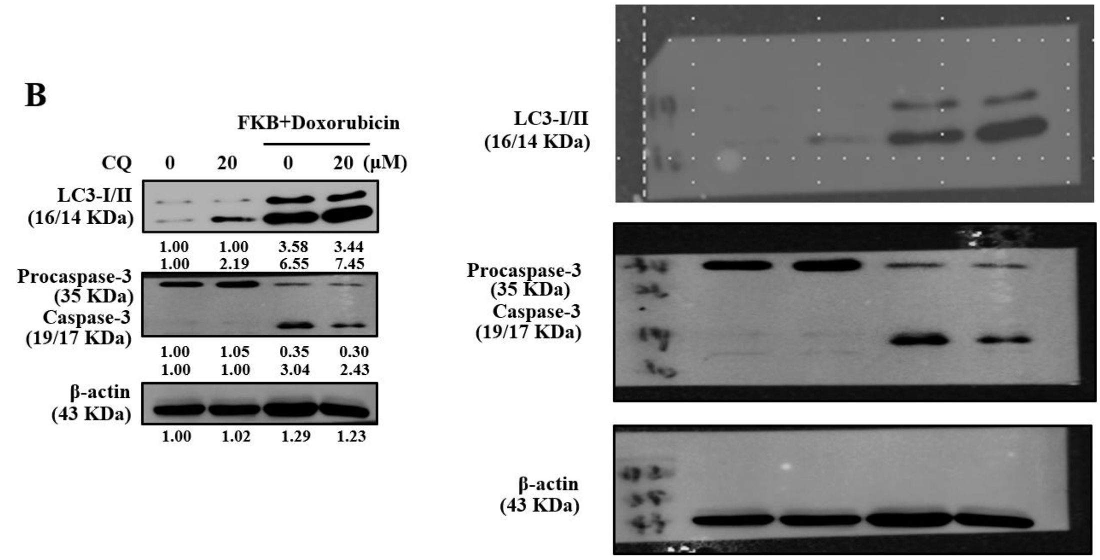
FKB+Doxorubicin
CQ 0 20 0 20 (μM)
LC3-I/II
(16/14 KDa)
1.00 1.00 3.58 3.44
1.00 2.19 6.55 7.45
Procaspase-3
(35 KDa)
Caspase-3
(19/17 KDa)
1.00 1.05 0.35 0.30
1.00 1.00 3.04 2.43
β-actin
(43 KDa)
1.00 1.02 1.29 1.23
LC3-I/II
(16/14 KDa)
Procaspase-3
(35 KDa)
Caspase-3
(19/17 KDa)
β-actin
(43 KDa)

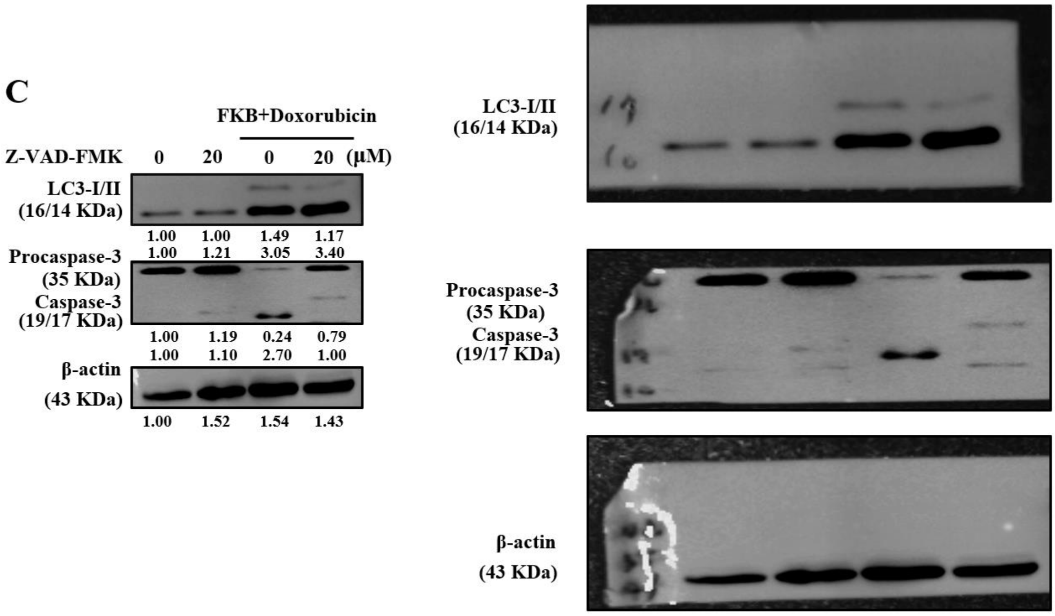

Figure S6. (**A–B**) Uncropped Western blot images of Inhibition of autophagy caused by FKB/doxorubicin suppressed apoptosis. Cells were pretreated with 3-MA (1 mM), related to Figure 7A–B. (**C**) Uncropped Western blot images of the monitored changes in the LC3-I/II and caspase-3 expressions, related to Figure 7C.

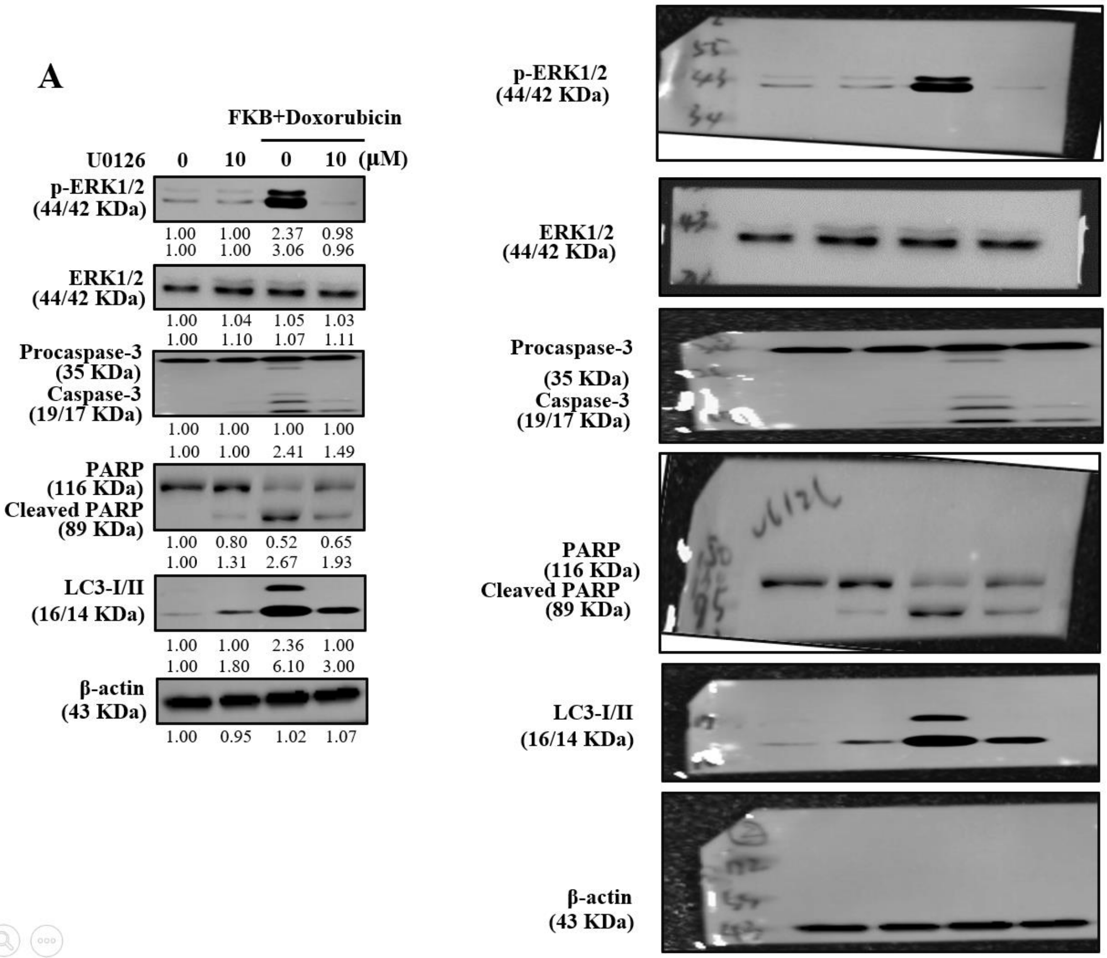

B

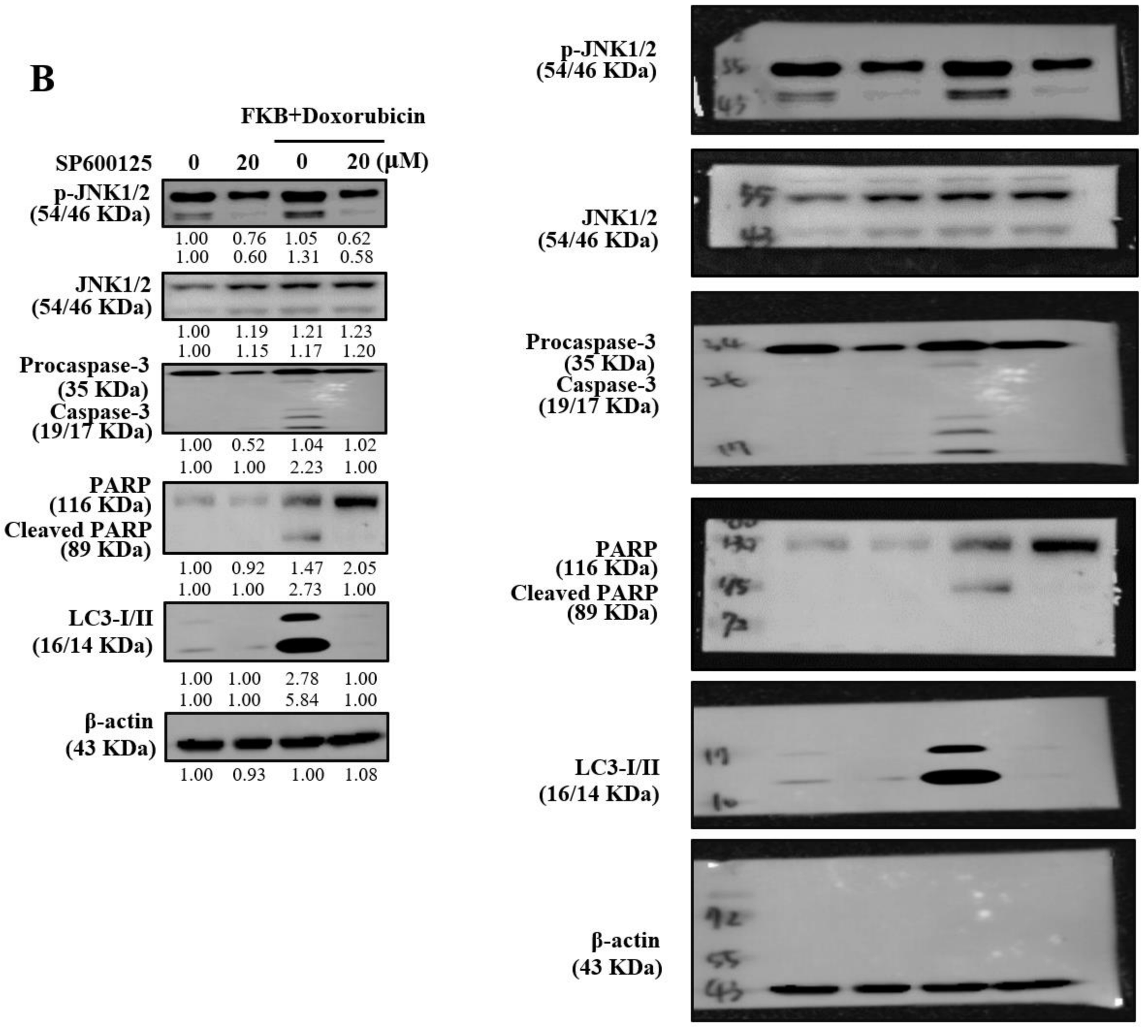

Figure S7. (**A**–**B**) Uncropped Western blot images of The effect of FKB and doxorubicin co-treatment on expression of LC3-I/II, caspase-3, and PARP was investigated and then changes in total and phosphorylated levels of ERK and JNK were assessed by Western blot, with caspase-3 and PARP expressions, related to Figure 8A–B.

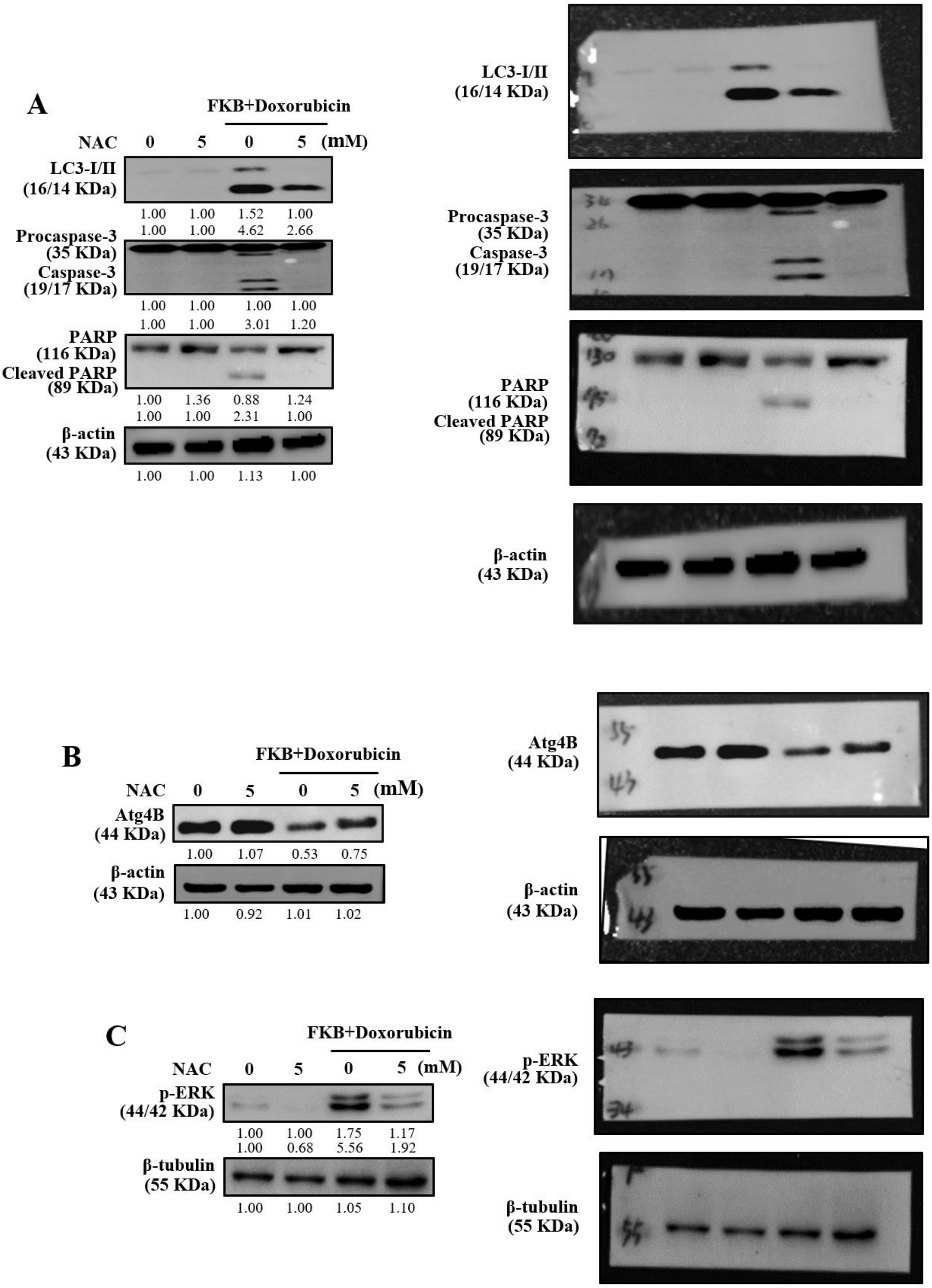

Figure S8. (**A**) Uncropped Western blot images of the terms caspase-3, PARP, and LC3-I/II, related to Figure 10A. (**B–C**) Uncropped Western blot images of The monitored for the terms ATG4B and p-ERK, related to Figure 10B–C.

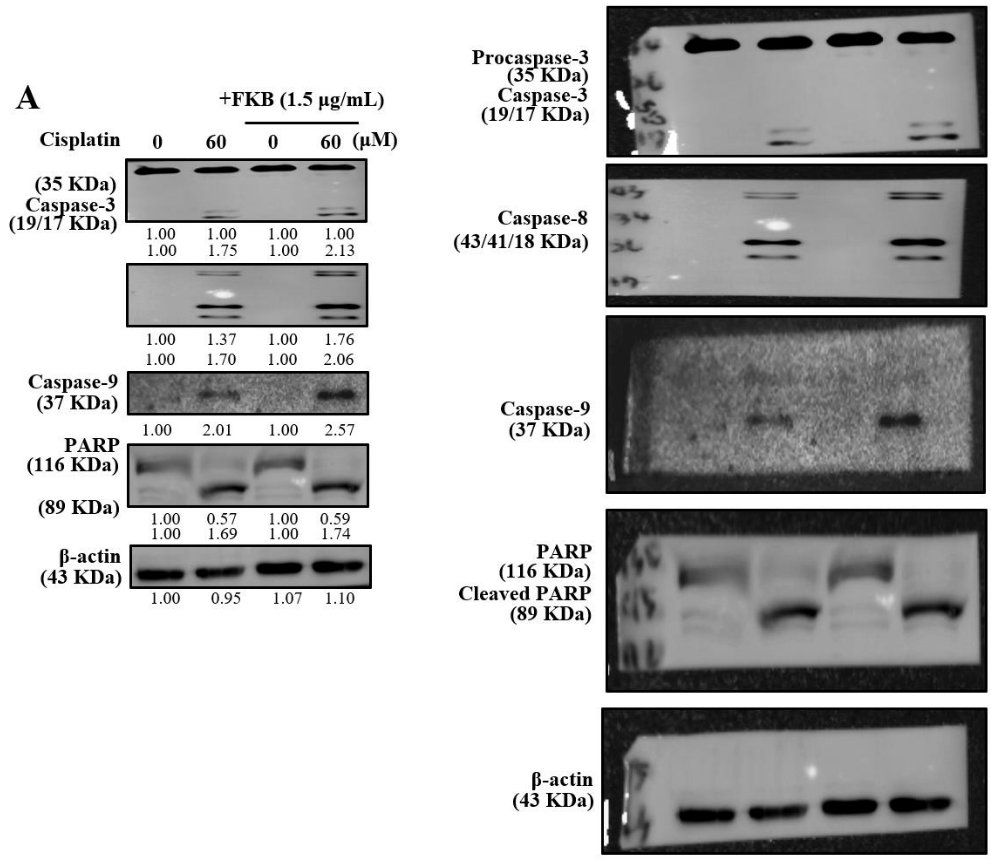

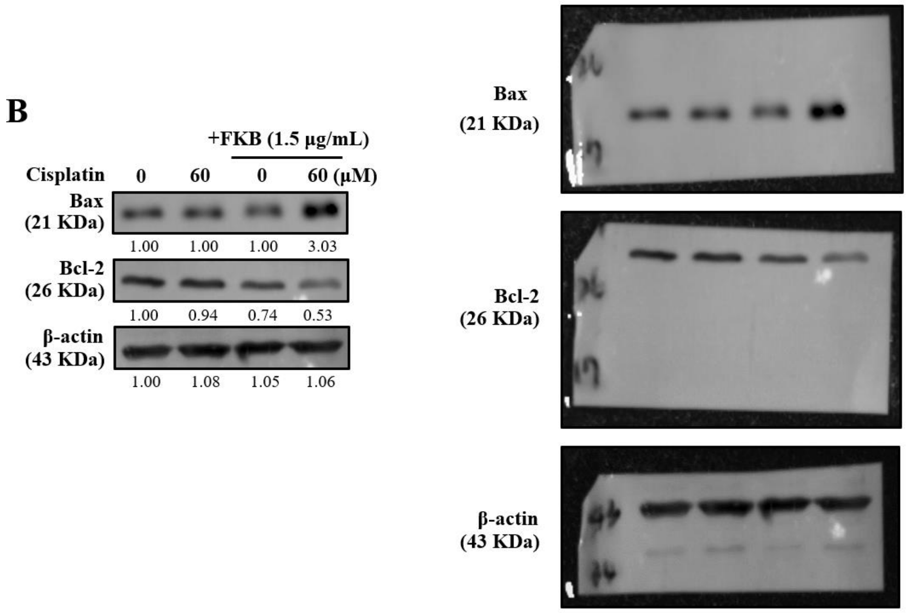

Figure S9. (**A**) Uncropped Western blot images of the the expression caspase-3, caspase-8, caspase-9, and PARP related to Figure 11D. (**B**) Uncropped Western blot images of The changes in the expression of Bax and Bcl-2 proteins, related to Figure 11E.
